# Transformer-based deep learning ensemble framework predicts autism spectrum disorder using health administrative and birth registry data

**DOI:** 10.1038/s41598-025-90216-8

**Published:** 2025-04-07

**Authors:** Kevin Dick, Emily Kaczmarek, Robin Ducharme, Alexa C. Bowie, Alysha L.J. Dingwall-Harvey, Heather Howley, Steven Hawken, Mark C. Walker, Christine M. Armour

**Affiliations:** 1 Better Outcomes Registry & Network (BORN) Ontario, Ottawa, Canada; 2 Prenatal Screening Ontario, Better Outcomes Registry & Network, Ottawa, Canada; 3 Children’s Hospital of Eastern Ontario Research Institute (CHEO-RI), Ottawa, Canada; 4 Clinical Epidemiology Program, Ottawa Hospital Research Institute, Ottawa, Canada; 5 School of Epidemiology and Public Health, University of Ottawa, Ottawa, Canada; 6 Department of Obstetrics and Gynecology, University of Ottawa, Ottawa, Canada; 7 ICES, Toronto, Canada; 8 International and Global Health Office, University of Ottawa, Ottawa, Canada; 9 Department of Obstetrics, Gynecology & Newborn Care, The Ottawa Hospital, Ottawa, Canada; 10 Department of Pediatrics, University of Ottawa, Ottawa, Canada; 11 Department of Genetics, CHEO, Ottawa, Canada

**Keywords:** Population screening, Paediatric research, Biomedical engineering, Computer science

## Abstract

Early diagnosis and access to resources, support and therapy are critical for improving long-term outcomes for children with autism spectrum disorder (ASD). ASD is typically detected using a case-finding approach based on symptoms and family history, resulting in many delayed or missed diagnoses. While population-based screening would be ideal for early identification, available screening tools have limited accuracy. This study aims to determine whether machine learning models applied to health administrative and birth registry data can identify young children (aged 18 months to 5 years) who are at increased likelihood of developing ASD. We assembled the study cohort using individually linked maternal-newborn data from the Better Outcomes Registry and Network (BORN) Ontario database. The cohort included all live births in Ontario, Canada between April 1st, 2006, and March 31st, 2018, linked to datasets from Newborn Screening Ontario (NSO), Prenatal Screening Ontario (PSO), and Canadian Institute for Health Information (CIHI) (Discharge Abstract Database (DAD) and National Ambulatory Care Reporting System (NACRS)). The NSO and PSO datasets provided screening biomarker values and outcomes, while DAD and NACRS contained diagnosis codes and intervention codes for mothers and offspring. Extreme Gradient Boosting models and large-scale ensembled Transformer deep learning models were developed to predict ASD diagnosis between 18 and 60 months of age. Leveraging explainable artificial intelligence methods, we determined the impactful factors that contribute to increased likelihood of ASD at both an individual- and population-level. The final study cohort included 707,274 mother-offspring pairs, with 10,956 identified cases of ASD. The best-performing ensemble of Transformer models achieved an area under the receiver operating characteristic curve of 69.6% for predicting ASD diagnosis, a sensitivity of 70.9%, a specificity of 56.9%. We determine that our model can be used to identify an enriched pool of children with the greatest likelihood of developing ASD, demonstrating the feasibility of this approach.This study highlights the feasibility of employing machine learning models and routinely collected health data to systematically identify young children at high likelihood of developing ASD. Ensemble transformer models applied to health administrative and birth registry data offer a promising avenue for universal ASD screening. Such early detection enables targeted and formal assessment for timely diagnosis and early access to resources, support, or therapy.

**Supplementary Information:**

The online version contains supplementary material available at 10.1038/s41598-025-90216-8.

## Introduction

Autism spectrum disorder (ASD) is a neurodevelopmental disorder characterized by enduring difficulties in social interaction, speech and nonverbal communication, and repetitive behaviors^1^. Individuals with ASD may be at increased risk for experiencing stressful and traumatic life events, the sequelae of which can negatively impact mental health through the development of comorbid psychopathology and/or worsening of the core symptoms of ASD^2,3^. Over the past two decades, the prevalence of ASD has notably risen: in 2018, approximately 1 in 44 US children were diagnosed with ASD by the age of 8 years^4^. Early diagnosis can greatly improve a child’s development^5,6^and help them realize their full potential, so they can access support and services as soon as possible. Early intensive interventions significantly improve behavioural and social outcomes for children with ASD. For example, applied behavior analysis therapy has been shown to enhance abilities like problem-solving and language skills; occupational therapy supports adaptive behaviors such as daily self-care activities; social skills training helps children better engage in peer interactions; and physical therapy improves motor skills, aiding in tasks like coordination and balance^7–9^. However, the diagnosis of ASD currently occurs via recognition of symptomology that is non-systematic and imprecise, resulting in missed and delayed diagnoses^10,11^. Despite the benefit of early identification of ASD, no universal screening programs exist. Prior research has identified a limited number of risk factors associated with ASD (e.g., complications at birth, family history of ASD, born to older parents, etc.), and routinely collected health data during pregnancy and early childhood offer an opportunity for universal screening and early diagnosis, intervention and support.

Routinely captured medical and health administrative data have been used to develop ASD screening algorithms. Rahman et al. combined maternal and paternal electronic medical records for 96,138 patients (1,397 ASD cases, 94,741 controls), and showed that models incorporating prescribed medications, parental age, and socioeconomic status to identify ASD achieved Area Under the Receiver Operating Characteristic curve (AUROC) values of 0.69–0.72^12^. Chen et al. used diagnostic and procedural codes from medical claims data for 38,576 individuals (12,743 ASD cases, 25,833 controls) to identify ASD in children of 18, 24, and 30 months old, with AUROC values between 0.71 and 0.87^13^. However, these studies only considered a limited number of features. Machine learning (ML) has been used to consider a wide range of features for identifying ASD cases among various age groups (e.g., toddlers, children, adolescents, and/or adults), including structural differences in brain magnetic resonance imaging (MRI)^14–16^, social and behavioural questionnaires^17–22^, and gene expression profiles^23,24^. These ML approaches, while promising, are not acceptable when leveraging certain types of data or feasible to acquire when applied across a population – for example ASD prediction based on genetic features alone could lead to stigmatization of families with a history of ASD, especially when applied to complete populations and with the risk of individuals being labeled inaccurately. To date, no predictive models for ASD screening have been developed and evaluated for population-level screening.

Deep learning (DL) algorithms are a class of dense artificial neural networks than can identify complex predictive features from vast volumes of data. DL models can mine comprehensive and granular individual-level records within these datasets and identify features that are most strongly associated with the outcome of interest. Transformer models are a new class of DL models that can be trained more efficiently than previous recurrent neural network architectures^25,26^. By also incorporating an explainable artificial intelligence (XAI) approach when training DL models, model developers and end users (e.g., healthcare practitioners) can gain insight into how various patient characteristics and other factors contribute to model predictions^27,28^. In this study, we examine the feasibility of using novel DL models and comprehensive, population-based, and routinely collected health data from Ontario to identify young children (up to 5 years of age) with elevated risk of developing ASD.

## Methods

We conducted a retrospective, population-based cohort study using data from ICES (“Institute for Clinical Evaluative Sciences”) - an Ontario-based independent, non-profit research institute whose legal status under Ontario’s health information privacy law allows it to collect and analyze health care and demographic data, without consent, for health system evaluation and improvement. ICES is designated a prescribed entity under Ontario’s Personal Health Information Privacy Act (PHIPA) and the Coroners Act.

All experimental protocols were approved by a named institutional and/or licensing committee. The Children’s Hospital of Eastern Ontario’s Research Ethics Board (REB# 22/06PE) and the ICES Privacy Office (ICES# 2023 901 377 000) approved this study. The Children’s Hospital of Eastern Ontario’s Research Ethics Board (REB# 22/06PE) and the ICES Privacy Office (ICES# 2023 901 377 000) waived the need for informed consent. The authors confirm that all research was performed in accordance with relevant guidelines & regulations.

This Study is based in part on data provided by Better Outcomes Registry and Network (“BORN”) Ontario, a prescribed registry under the Personal Health Information Protection Act^29^that contains routinely collected data for pregnancies, births, and newborns across the province of Ontario since 2012^30^. As a registry, BORN is afforded the authority to collect personal health information (PHI) without consent for such purposes subject to PHIPA, its regulation (O. Reg. 329/04, available at https://www.ontario.ca/laws/regulation/040329) and procedures approved of by the information and Privacy Commissioner of Ontario. Parts of this material are based on data and/or information compiled and provided by CIHI and the Ontario Ministry of Health.

**Fig. 1:**
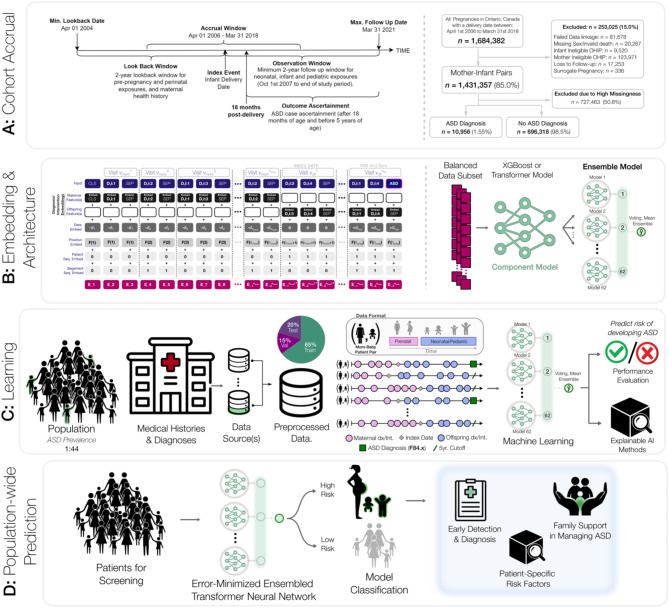
Training and prediction of autism spectrum disorder from mother-offspring health data. **A**, Cohort Accrual: we specify the health data leveraged within our study and their inclusion/exclusion criteria. The maternal look-back window relative to the index is a maximum of 2 years and the offspring observation window is a minimum of 2 years and up to a maximum of 5 years. The outcome of interest is an ASD diagnosis between 18 and 64 months. **B**, Embedding & Architecture: the trained model requires converting real-world clinical data into an embedding – a transformation of categorical disease and intervention codes, timestamps, and related patient data into a lower-dimensional real number continuous space. The transformer model then extracts relevant patterns from the disease history and leverages this latent space to generate ASD risk predictions. To make use of all available data, we trained 62 individual component models and combined their predictions within a large-scale voting ensemble model that outputs a final high-confidence prediction. **C**, Learning: the general ML framework begins by portioning the mother & offspring medical histories into a training set, a validation set, and a test set. The data sources are numerous linked repositories aggregated by mother-offspring ID and preprocessed into both time-series and static one-hot encoded representations for subsequent machine learning algorithm development. The training and validation datasets are used to train the models and minimize the prediction error. **D**, Population-wide Prediction: we evaluate the final model’s prediction performance on the independently held-out test set to quantify its ability to generalize to unseen cases. The term “error-minimized model” was intentionally used to emphasize the optimization process aimed at reducing prediction error during model training. This final model is used to discriminate between patients at higher and lower risk of developing ASD and this risk model can be leveraged as part of a population screening program.

We implemented two distinct ML algorithms, Transformer and Extreme Gradient Boosting (XGBoost)^25,26,31^, using individual-level mother-infant health data to develop and internally validate a predictive model, leveraging XAI methods to identify features associated with developing ASD. An overview of our methodological framework is presented in Fig. 1. The analyses, conclusions, opinions and statements expressed herein are solely those of the authors and do not reflect those of the funding or data sources; no endorsement is intended or should be inferred.

### Data acquisition and outcome definition

This study combines both maternal (prenatal) and offspring (fetal & postnatal) characteristics for ASD prediction (Fig. 1). Maternal characteristics and medical information prior to and during pregnancy were considered with a look-back period of two years from the offspring’s date of birth (Fig. 1C). A follow-up period was applied to collect offspring characteristics, beginning at birth and continuing for five years, until ASD was diagnosed, or until the last available data entry in ICES - whichever occurred first (Fig. 1A). All datasets were linked using unique encoded identifiers and analyzed at ICES. Our cohort was derived from Better Outcomes Registry & Network (BORN) Ontario: a provincial prescribed perinatal, newborn and child registry^1^. The cohort consisted of all live births between April 1st, 2006 – March 31st, 2018, with mother and offspring information linked through the MOMBABY dataset at ICES. This cohort was then linked to additional datasets: Newborn Screening Ontario (NSO), Prenatal Screening Ontario (PSO), Canadian Institute for Health Information (CIHI)’s Discharge Abstract Database (DAD), and CIHI’s National Ambulatory Care Reporting System (NACRS). NSO and PSO datasets contain screening biomarker values and outcomes, whereas DAD and NACRS consist of International Classification of Diseases (ICD-10) diagnostic codes and Canadian Classification of Health Intervention (CCI) intervention codes assigned during hospital and/or emergency visits and outpatient surgeries. Pairs were excluded from the study cohort using the following criteria: failed linkage to other datasets, invalid death dates, missing offspring sex, mothers or offspring ineligible for the Ontario Health Insurance Plan (OHIP) coverage during the entire study period, offspring with missing follow-up information, and offspring resulting from surrogate pregnancies. Any offspring with missing gestational age or birth weight, or with an ASD diagnosis after 5 years of age were also removed. Next, the cohort was limited to births between 2012 and 2018 due to high levels of missingness of key biomarkers included in NSO and PSO before 2012. Records with more than 50% missing NSO and PSO data, and those without at least one health contact in the DAD/NACRS datasets were removed (Fig. 1A).

The primary outcome of interest was diagnosis of ASD between 18 months to 5 years of age. ASD status was ascertained by a case-finding algorithm previously validated in Ontario health administrative data, which assigns a diagnosis of ASD for all those with at least one F84.x ICD-10 diagnostic code within their records from a hospital discharge, emergency department visit, or outpatient surgery, or the OHIP diagnostic code 299.x a minimum of 3 times in 3 years)^32^. This algorithm was clinically validated and found to have sensitivity of 50.0%, specificity of 99.6%, positive predictive value of 56.6% and a negative predictive value of 99.4%^32^.

### Descriptive analyses

Descriptive analyses were conducted to compare characteristics of mother-offspring pairs with an ASD diagnosis to those without (Table 1). Continuous variables were described using means (SDs) or medians (IQRs). Categorical variables were described using frequencies, percentages and standardized mean differences (SMDs).

**Table 1:** Complete cohort descriptive statistics. Definitions of each variable listed can be found in the supplementary appendix.

	Non. Cases	ASD. Cases	SMD
	(*N* = 696,318)	(*N* = 10,956)	
**Maternal Age at Delivery**			
Mean (SD)	30.7 (5.3)	31.0 (5.6)	0.0544
Median (IQR)	31.0 (27.0, 34.0)	31.0 (27.0, 35.0)	*N/A*
**Pre-Pregnancy BMI**			
Mean (SD)	21.3 (11.5)	21.2 (12.4)	0.0095
Median (IQR)	23.0 (19.5, 27.4)	23.1 (18.8, 28.2)	*N/A*
**Pre-Pregnancy BMI Category**			
Underweight	29,299 (4.2%)	539 (4.9%)	0.0354
Normal	298,843 (42.9%)	4111 (37.5%)	0.1090
Overweight	140,004 (20.1%)	2179 (19.9%)	0.0054
Class I Obesity	63,975 (9.2%)	1141 (10.4%)	0.0424
Class II Obesity	28,010 (4.0%)	522 (4.8%)	0.0377
Class III Obesity	20,855 (3.0%)	409 (3.7%)	0.0432
Missing	115,332 (16.6%)	2055 (18.8%)	0.0590
**Maternal Pre-Existing Health Condition (Any)**			
Yes	132,274 (19.0%)	2466 (22.5%)	0.0894
No	522,610 (75.1%)	7781 (71.0%)	0.0931
Missing	41,434 (6.0%)	709 (6.5%)	0.0220
**Maternal Diabetes Diagnosis**			
Yes	52,330 (7.5%)	1227 (11.2%)	0.1393
No	637,132 (91.5%)	9632 (87.9%)	0.1282
Missing	6856 (1.0%)	97 (0.9%)	0.0101
**Maternal Hypertension Diagnosis**			
Yes	37,043 (5.3%)	809 (7.4%)	0.0917
No	650,566 (93.4%)	9984 (91.1%)	0.0927
Missing	8709 (1.3%)	163 (1.5%)	0.0213
**Maternal Pre-Existing Heart Disease**			
Yes	14,918 (2.1%)	289 (2.6%)	0.0342
No	639,966 (91.9%)	9958 (90.9%)	0.0372
Missing	41,434 (6.0%)	709 (6.5%)	0.0220
**Maternal Pre-Existing Asthma**			
Yes	26,922 (3.9%)	521 (4.8%)	0.0460
No	627,962 (90.2%)	9726 (88.8%)	0.0473
Missing	41,434 (6.0%)	709 (6.5%)	0.0220
**Maternal ASD Diagnosis**			
No	654,884 (94.0%)	10,247 (93.5%)	0.0220
Missing	41,434 (6.0%)	709 (6.5%)	*N/A*
**Maternal Family History of ASD**			
Yes	85 (0.0%)	1–5	* N/A*
No	3714 (0.5%)	79 (0.7%)	0.0257
Missing	692,519 (99.5%)	10,872–10,876	* N/A*
**Maternal Mental Health Disorder (Any)**			
Yes	108,585 (15.6%)	2239 (20.4%)	0.1332
No	546,669 (78.5%)	7972 (72.8%)	0.1397
Missing	41,064 (5.9%)	745 (6.8%)	0.0383
**Maternal Mood Disorder**			
Yes	56,083 (8.1%)	1274 (11.6%)	0.1309
No	599,171 (86.0%)	8937 (81.6%)	0.1290
Missing	41,064 (5.9%)	745 (6.8%)	0.0383
**Maternal Anxiety Disorder**			
Yes	61,666 (8.9%)	1291 (11.8%)	0.1028
No	593,588 (85.2%)	8920 (81.4%)	0.1078
Missing	41,064 (5.9%)	745 (6.8%)	0.0383
**Maternal Psychotic Disorder**			
Yes	487 (0.1%)	21 (0.2%)	0.0454
No	654,767 (94.0%)	10,190 (93.0%)	0.0432
Missing	41,064 (5.9%)	745 (6.8%)	0.0383
**Maternal Neurodevelopmental Disorder (Any)**			
Yes	163 (0.0%)	1–5	* N/A*
No	654,721 (94.0%)	10,243 (93.5%)	0.0225
Missing	41,434 (6.0%)	707–711	* N/A*
**Paternal ASD Diagnosis**			
Yes	1–5	0 (0.0%)	*N/A*
No	3279 (0.5%)	69 (0.6%)	0.0231
Missing	693,034–693,038	10,887 (99.4%)	*N/A*
**Paternal Family History of ASD**			
Yes	74 (0.0%)	1–5	* N/A*
No	3107 (0.4%)	63 (0.6%)	0.0193
Missing	693,137 (99.5%)	10,890–10,894	* N/A*
**Smoking During Pregnancy**			
Yes	71,349 (10.2%)	1443 (13.2%)	0.0962
No	598,668 (86.0%)	8961 (81.8%)	0.1203
Missing	26,301 (3.8%)	552 (5.0%)	0.0660
**Drug Use During Pregnancy (Any)**			
Yes	14,893 (2.1%)	354 (3.2%)	0.0752
No	641,529 (92.1%)	9848 (89.9%)	0.0832
Missing	39,896 (5.7%)	754 (6.9%)	0.0495
**Alcohol Use During Pregnancy**			
Yes	15,155 (2.2%)	200 (1.8%)	0.0241
No	639,983 (91.9%)	9935 (90.7%)	0.0450
Missing	41,180 (5.9%)	821 (7.5%)	0.0668
**Conception Type**			
Assisted	28,069 (4.0%)	538 (4.9%)	0.0446
Spontaneous	633,578 (91.0%)	9794 (89.4%)	0.0557
Missing	34,671 (5.0%)	624 (5.7%)	0.0329
**Mode of Delivery**			
Caesarean Section	200,921 (28.9%)	3852–3856	* N/A*
Vaginal	495,300 (71.1%)	7099 (64.8%)	0.1397
Missing	97 (0.0%)	1–5	* N/A*
**Type of Labour**			
Induced	176,918 (25.4%)	3026 (27.6%)	0.0508
Spontaneous	412,361 (59.2%)	6026 (55.0%)	0.0858
No Labour	106,673 (15.3%)	1894 (17.3%)	0.0546
Missing	366 (0.1%)	10 (0.1%)	0.0168
**Pain Management at Delivery**			
Neuraxial Anesthesia	402,646 (57.8%)	6774 (61.8%)	0.0811
Other	96,331 (13.8%)	1194 (10.9%)	0.0852
No	89,099 (12.8%)	1081 (9.9%)	0.0878
Missing	108,242 (15.5%)	1907 (17.4%)	0.0513
**Gestational Age at Delivery (weeks)**			
Mean (SD)	38.8 (2.1)	38.4 (2.5)	0.1846
Median (IQR)	39.0 (38.0, 40.0)	39.0 (38.0, 40.0)	*N/A*
**Birth Weight (grams)**			
Mean (SD)	3347.1 (568.4)	3262.2 (644.7)	0.1491
Median (IQR)	3370.0 (3030.0, 3703.0)	3305.0 (2930.0, 3665.0)	*N/A*
**Infant Sex**			
Male	354,219 (50.9%)	8424 (76.9%)	0.5216
Female	342,099 (49.1%)	2532 (23.1%)	*N/A*
**Birth Season**			
Spring	175,795 (25.2%)	2699 (24.6%)	0.0141
Summer	189,029 (27.1%)	3071 (28.0%)	0.0199
Fall	169,304 (24.3%)	2793 (25.5%)	0.0275
Winter	162,190 (23.3%)	2393 (21.8%)	0.0343
**Intention to Breastfeed**			
Yes	606,131 (87.0%)	9161 (83.6%)	0.1020
No	42,692 (6.1%)	823 (7.5%)	0.0575
Missing	47,495 (6.8%)	972 (8.9%)	0.0812
**Apgar Score at 5 min**			
Normal	686,836 (98.6%)	10,775 (98.3%)	0.0250
Low	3230 (0.5%)	82 (0.7%)	0.0417
Missing	6252 (0.9%)	99 (0.9%)	0.0006
**NICU Admission**			
Yes	93,715 (13.5%)	2221 (20.3%)	0.1990
No	602,603 (86.5%)	8735 (79.7%)	*N/A*

### Data preprocessing

Due to the different strategies required for processing temporal and static data from multiple sources, datasets were preprocessed separately according to distinct protocols (Fig. 1B, details in Online Supplement). These datasets were linked using unique encoded identifiers and analyzed at ICES.

### Machine learning

We implemented and evaluated two ML algorithms for the prediction of ASD: BEHRT, a transformer developed for the analysis of electronic health records (EHR)^33^, and XGBoost a boosted tree-based ML model^34^. BEHRT is a time-series model that analyzes temporal sequences of hospital visits and matching sequences comprising the date of the visit and the patient admitted. These sequences are embedded into latent representations and combined as the input to transformer attention layers, with a final linear layer for prediction of ASD (Fig. 1B). To compare performance to that of a non-DL baseline method, we transformed temporal variables into static representation and predicted ASD with XGBoost. We pretrained BEHRT with masked-language-modelling and used the architecture from the original publication that delivered the best performance^33^. For XGBoost, we applied our previous work, and ran large-scale hyperparameter tuning experiments leveraging high-performance computing infrastructure^35^. See Supplementary Appendix for complete details.

Following methodology that is standard within ML research, the data were divided into training, validation, and test partitions with a respective 65:15:20% split, stratifying both by outcome (ASD vs. non-ASD) and maternal identifier (maternal/parental features only appear within independent sets) (Fig. 1C). This two-part stratification ensures equal representation of ASD and prevents data leakage of the same mother appearing in both data partitions. Any transformations applied to numeric variables were first performed on training data; the same parameters were then used to transform the validation and test data.

Training individual models with balanced training data (downsampling the majority class to 1:1) would result in the loss of 469,051 of non-ASD cases, limiting the generalizability of our findings. We used large-scale ensemble of component models for our final model, where each model was trained with the same 7,624 of ASD cases and a different subset of non-ASD controls (randomly sampled without replacement), ultimately leveraging all available data. The validation data was leveraged as part of hyperparameter tuning our models and comparing results across experiments. The testing data was then evaluated on each model, and majority voting was used for final ASD prediction. This approach was applied to both the BEHRT and XGBoost model architectures.

### Evaluation metrics

**Fig. 2:**
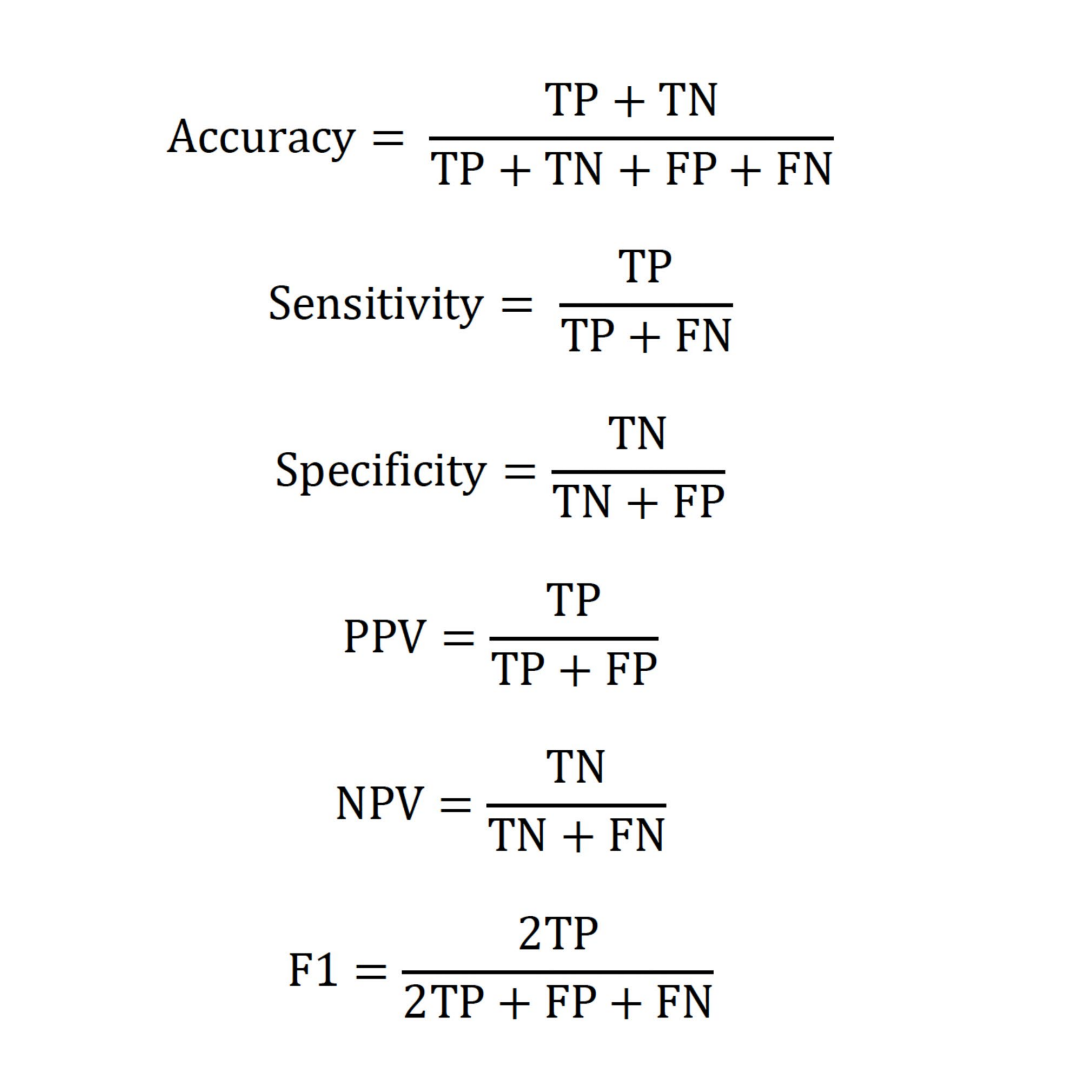
Performance metrics used for all models. TP: true positives (number of instances correctly predicted to be positive); TN: true negatives (number of instances correctly predicted to be negative), FP: false positives (number of instances incorrectly predicted to be positive); FN: false negatives (number of instances incorrectly predicted to be negatives).

We assessed prediction performance, for all models trained and evaluated, by measuring sensitivity (i.e., true positive rate or recall), specificity (i.e., true negative rate), and positive predictive value (PPV or precision) at different risk thresholds (Fig. 2). As depicted in Fig. 1D, an error-minimized model could be leveraged in the future as a component of a population screening program. The model’s overall ability to discriminate was determined using the area under the receiver operating characteristic curve (AUROC). Given the extreme class imbalance, we additionally report the F1 score (defined as the harmonic mean of PPV and sensitivity) and the area under the precision-recall curve (AUPRC). Finally, we reported the specificity and PPV value where the predicted probability cutoff yields a sensitivity of 50%. Cumulative gain curves were also created to express that our model can be used to identify an enriched pool of high-risk children up to the age of 5.

### Algorithm explainability

To identify the most predictive features for ASD risk, we used game-theoretic SHapley Additive exPlanations (SHAP) analysis^36^to query the trained models and obtain an indication of how significant each factor is in determining the final ASD prediction^37^. SHAP analysis generates many prediction experiments that vary ‘coalitions’ (or feature combinations) to compare the impact of variable inclusion/exclusion against the other features to quantitatively assess the average impact of a given feature on the overall model^36^.

## Results

The final study cohort included 707,274 mother-infant pairs, from deliveries between 2012 and 2018, including 10,956 ASD cases (1.55%) (Fig. 1A). We observed imbalance between outcome groups in several maternal and infant characteristics (Table 1). Compared to children without ASD, more children with ASD were male, were delivered by caesarean section, were admitted to the NICU, had a lower mean birth weight, and were younger gestational age at delivery. In addition, pre-existing diagnoses of maternal mental health disorders or diabetes were more prevalent in mothers to children with ASD, compared to those to children without ASD. Reported smoking during pregnancy was also more prevalent in mothers of children with ASD.

Table 2 lists the results of the hyperparameter tuning experiments and final model performance. Resampling experiments revealed that the highest performance (using high sensitivity as the objective) was achieved from downsampled balanced ASD and non-ASD cases during training, motivating the development of a large-scale ensemble model. The final best-performing ensemble model achieved an AUROC of 69.6%, a sensitivity of 70.9%, a specificity of 56.9%, a positive predictive value of 2.4%, and a negative predictive value of 99.22%.

**Table 2:** Hyperparameter tuning transformer models on the validation dataset & evaluating generalizability on the test datasets. Final model selected by highest sensitivity on the validation dataset (bold). * indicates the *mean* metric of the ensembled models rather than the *voting ensemble* metric. AUROC refers to the model’s “Area under the receiver operating Characteristic” curve metric, LR refers to the model’s “Learning Rate”, PPV refers to the model’s “Positive predictive Value”. Pr@50Re metric reports the precision of a model when it achieves 50% recall while the Sp@50Se metric reports the specificity of a model when it reaches 50% sensitivity.

Experiment	Exp. Param.	Sensitivity	Specificity	Accuracy	AUROC	PPV	NPV	F1 Score
Ensembled Models	Grouped Codes	60.10%	61.90%	61.90%	65.50%*	2.50%	*N/A*	4.70%
	All Codes	61.80%	60.80%	60.80%	66.10%*	2.50%	*N/A*	4.70%
	Pretrained	68.10%	61.40%	61.50%	70.90%*	2.70%	*N/A*	5.30%
	Pretrained w/ LR decay	72.00%	57.90%	58.10%	71.20%*	2.70%	*N/A*	5.10%
	**Pretrained w/ LR decay**,** batch size = 64**	**72.30%**	**58.00%**	**58.20%**	71.10%*	**2.70%**	**99.24%**	**5.20%**
*Final Model; Test Dataset*	Pretrained w/ LR decay, batch size = 64	70.90%	56.90%	57.10%	69.60%*	2.40%	99.22%	4.70%

Validation Dataset: Pr@50Re: 3.2%; Sp@50Se: 76.2%.

Test Dataset: Pr@50Re: 2.8%; Sp@50Se: 73.5%.

**Fig. 3:**
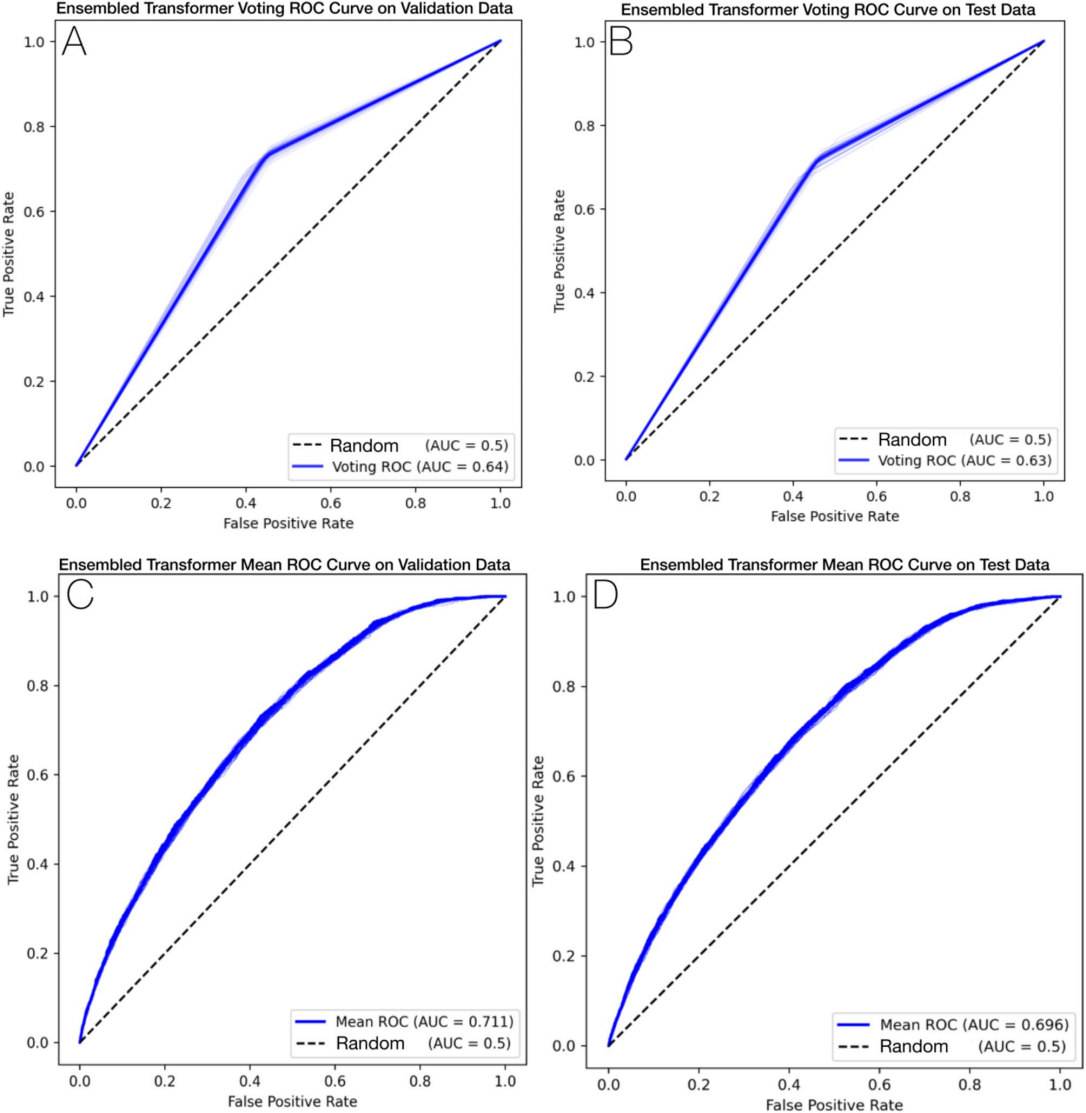
ROC curve summarizing performance of the final ensembled transformer model on the validation and test datasets. All *n* = 62 individual component model curves are plotted in light blue, overlaid but the voting (A, B) and mean (C, D) ROC curves summarizing the overall performance with respect to the validation (A, C) and test (B, D) datasets.

**Fig. 4:**
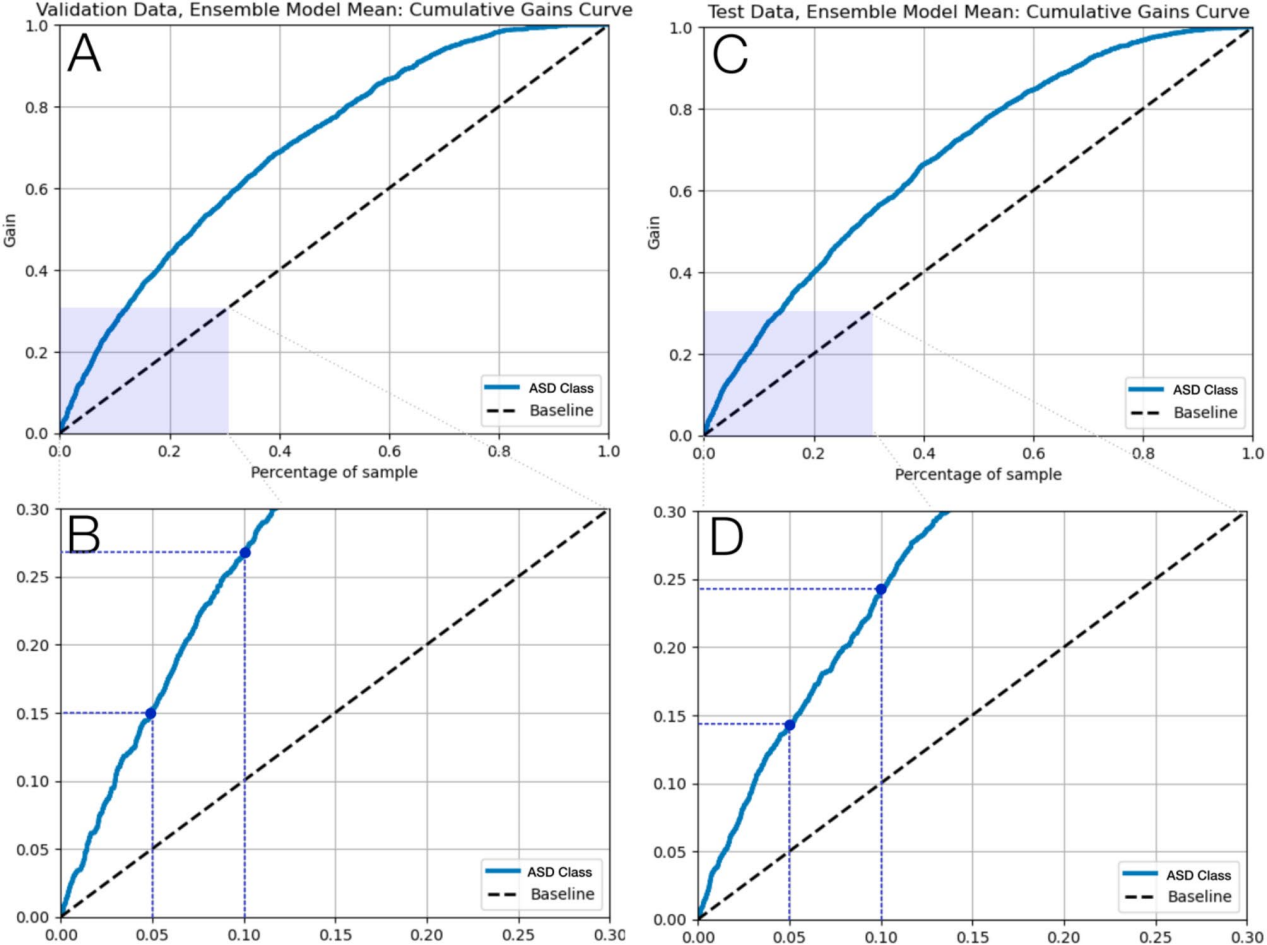
Ensemble transformer mean model cumulative gain curves.

The receiver operator characteristic (ROC) curves for the voting and mean ensemble Transformer model for validation and test datasets are illustrated in Fig. 3. Consistent performance curves across the validation and test datasets indicates our model did not overfit the training data and generalizes well to unseen data. From the cumulative gain plots in (Fig. 4), we note that the top-5% of the model’s prediction contain approximately 15% of all true cases and the top-10% of model predictions contain 25% of all true cases, suggesting that our model can be used to identify an enriched pool of high-risk cases.

**Fig. 5:**
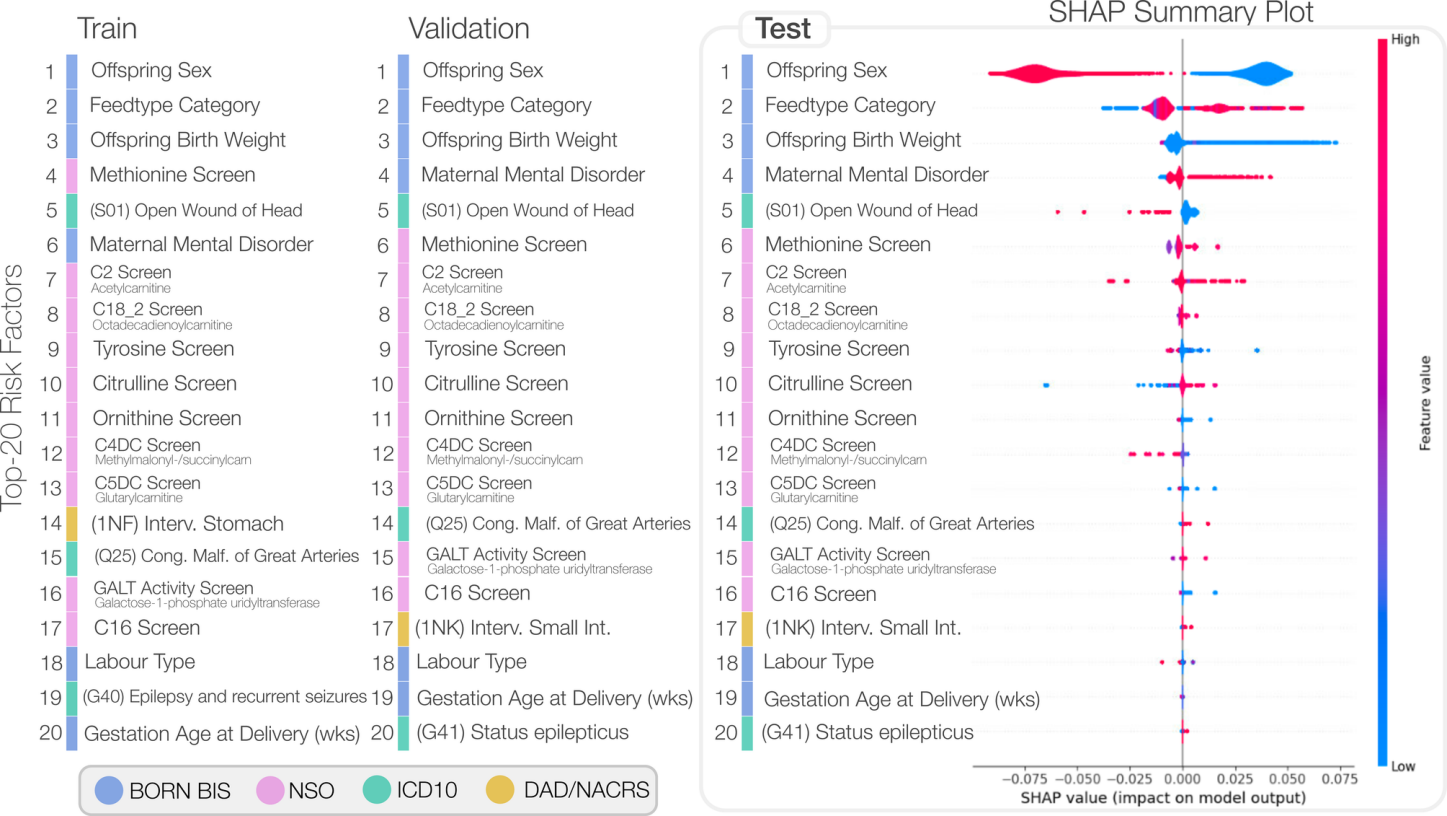
Summary of the top-ranking risk factors determined using SHAP analysis across the three independent datasets. The right-most SHAP summary plot depicts the violin plot distribution for each factor. The possible feature values for each variable are tabulated in Supplementary Table S3; individual KDE plots comparing ASD and controls are illustrated in Supplementary Figure S2. Both the C2 and C16 screen for acetylcarnitine analytes.

An illustration of the top-ranking features identified via SHAP analysis across the three independent datasets is presented in Fig. 5, where a positive SHAP value suggests that the factor increases the predicted probability of ASD while a negative value suggests that the feature decreases the predicted probability of ASD. Interestingly, a mixture of BORN-BIS, NSO, ICD-10, and DAD/NACRS features rank among the top-20 of each set with a general consistency.

## Discussion

In this retrospective, population-based cohort study from Ontario, Canada, we designed and implemented ML models to predict ASD in our final study cohort of 703,894 mother-offspring pairs. The best-performing ensemble of Transformer models achieved an AUROC of 69.6% for predicting ASD diagnosis, a sensitivity of 70.9%, a specificity of 56.9%; results that are consistent with similar studies such as the work of Betts et al.^38^. We applied ML best practices for training a predictor of ASD: comprehensive evaluation metrics, stratified train-test splits, and robust models that address class imbalance. We demonstrated model generalizability given that both Figs. 3 and 4 illustrate similar performance across validation and test datasets and Fig. 5 shows that the most impactful model features and ordering are also consistent across train, validation, and test datasets.

The results of this work demonstrate feasibility and potential to identify young children with increased likelihood of developing ASD using a ML model applied to population-based and routinely collected data. The models presented within this work also have demonstrated face validity given that top-ranking predictive features (Fig. 3) include known ASD risk factors (e.g., male sex, low birth weight). By incorporating large-scale and heterogenous datasets, these models and XAI features provide testable hypotheses for future work. Our models highlight a number of newborn screening factors that, following additional investigation, could be incorporated within an early life universal ASD screening program.

To our knowledge, the only other studies to have used EMR ICD codes and a similar ML methodology were conducted by Betts et al. and Bishop-Fitzpatrick et al.^38,39^. Our work reveals significant advances over these studies. The Betts et al. dataset ranged from 2003 to 2005 with approximately 260,000 offspring^39^, whereas our work includes ~ 700,000 offspring and spans 2012–2018. While earlier models utilized Logistic Regression and XGBoost models^39^, we apply Transformer-based models^40^ that sequentially analyze mother-infant medical histories. Regarding performance, our work achieves a slightly lower AUROC score of 70% compared to the 73% reported by Betts et al.^39^. The fact that we demonstrate similar performance is significant given that these studies originate from completely different international settings. The complementary use of SHAP analysis^41,42^as an XAI method within both studies offer promising insights into the candidate factors that may assist healthcare providers in understanding this complex neurodevelopmental condition. Finally, our use of a large-scale ensembling model architecture to address extreme class imbalance typical within healthcare dataset (as well as our approach to extensive hyperparameter tuning^43,44^) are notable contributions that advance conventional applied healthcare ML methodologies.

One of the limiting factors of all studies using medical claims for prediction of ASD, including ours, is the increased number of hospital/doctor’s visits that ASD patients have compared to normal cases. This may introduce bias into the data, where the ML model begins to make predictions based on the number of visits a child has, as opposed to true ASD risk factors. While this is inherent in any medical claims data, our study mitigates this risk by combining offspring visits with maternal visits and padding/truncating all visits/codes to a length of 200. In addition, we choose to include the entire cohort of non-ASD controls, as opposed to selecting a subset for model training and evaluation. Although we have a similar number of total ASD cases to Chen et al.^13^., our model can better generalize to the entire population for screening ASD due to the inclusion of all possible population data. Other limitations include the lack of paternal information, imposing a bias and unequitable focus on maternal factors. Unfortunately, paternal medical data is not reliably collected and should be the subject of future research.

Another important limiting factor of this work originates from the ICES ASD algorithm from which the ASD ground truth labels are acquired. The work of Brooks et al. assessed numerous algorithms for the identification of children with ASD in health administrative datasets resulting in the labels leveraged in this work^32^. Their optimal algorithm achieved a sensitivity of 50.0% (95% CI 40.7–88.7%), specificity of 99.6% (99.4–99.7), PPV of 56.6% (46.8–66.3), and NPV 99.4% (99.3–99.6)^32^. Given the performance of this algorithm in establishing the ground truth for ASD in our own study, we cannot expect the models produced herein to exceed this level of performance.

Bias must be considered before deploying any clinical decision-support tool that incorporates ML for early detection. Biases can originate in the data used, the algorithm, or a combination of both. For instance, in our study focusing on childhood ASD, the manifestation of the condition prevalence differs significantly between males and females (sex assignment at birth). This discrepancy leads to a lower rate of diagnosis and, consequently, a reduction in available treatment in female patients. If not fully considered and transparently understood, models like ours could unintentionally exacerbate this gender disparity. Our model may result in increased likelihood of misclassification among females, erroneously classifying females with ASD as controls in the sample data. Additionally, the skewed representation of male patients in the training data may unintentionally cause the algorithm to optimize for male-related indicators, thereby enhancing prediction accuracy for males while diminishing it for females. To prevent such discriminatory outcomes, it is imperative to thoroughly evaluate model performance across different patient subgroups and implement measures to mitigate these biases.

Accounting of temporal biases is an important consideration, as this work includes a broad representation of health administration and registry data spanning the prenatal, perinatal, postnatal, and pediatric periods. Given the need for large-scale datasets to train our transformer-based model, this work aggregated data from conception to up to 5 years postnatal to determine whether ASD could be accurately predicted from this accumulation of evidence. We recognize that certain factors occurring at a later stage of development may be more predictive of ASD, as illustrated among the top-ranking factors from our SHAP analysis (Fig. 5), and this may be indicative of a temporal bias where individuals diagnosed later in childhood may be more easily detected by our method than individuals who may not have equally represented health care data. Moreover, the prevalence of ASD in the population has increased over time, implying that our model may be biased to detecting individuals with more recent health care records. Finally, data quality may change over time. We report the standard deviation of our model performance across birth year strata in the supplementary materials and note that, while some variation exists in trading off performance (specifically between model sensitivity and specificity), performance is largely consistent. However, future work should investigate optimizing model training for such cross-year analyses.

For this study, we focused on identifying broader patterns of prediction and the overall robustness of a model developed from a large-scale representation of the population. Nonetheless, we recognize the importance of developing models that account for time-varying factors and future work will examine their impact on model performance. For example, we will discern the model impact of early/late ASD detection by widening (> 5 years) or narrowing (< 5 years) the window of time considered for maximum follow-up. To investigate model performance in accordance to the quality of data available at specific snapshots in time, future research will also consider stratification by year of birth as part of a leave-one-year-out cross-validation approach for model training.

Additional future work will seek to improve performance and identify new datasets for predicting ASD via our framework. Universal screens must limit the number of features considered by the model to those most reliable and impactful to consistently distinguishing ASD in young children. Thus, future will work will also apply an ablation-like approach to ensure that we develop and implement a model for which the data is reliably collected, and the clinical relevance of the features are well-understood. Ultimately, such an AI-based ASD screen will have the potential for deployment and use as a clinical decision support system, as depicted in Fig. 1D. Additionally, the methodology and framework developed within this study could be applied to other complex neurodevelopmental conditions, towards a multi-condition screening framework.

## Conclusion

This study demonstrates the feasibility of applying ML models to population-based and routinely collected health information to systematically identify young children who are likely to develop ASD. Evaluated on a fully independent dataset representative of a general population sample, our model’s reported sensitivity of 70.9%, specificity of 56.9%, and AUROC of 69.6% suggest that our ensemble transformer model is a promising candidate for population-based ASD screening. Early identification through this method could facilitate comprehensive and timely assessment for ASD, ensuring prompt diagnosis and faster access to resources, support, or therapy.

## Electronic supplementary material

Below is the link to the electronic supplementary material.

Supplementary Material 1

## Data Availability

The Children’s Hospital of Eastern Ontario’s Research Ethics Board (REB# 22/06PE) and the Institute of Clinical Evaluative Sciences (ICES) Privacy Office (ICES# 2023 901 377 000) approved this study. This study was based on data compiled by ICES and on data and/or information compiled and provided by CIHI and so are not publicly available. However, the analyses, conclusions, opinions, and statements expressed herein are those of the author(s), and not necessarily those of ICES or CIHI. The datasets generated and/or analysed during the current study are not publicly available due to the individual risk for re-identification, but are available upon reasonable request from the corresponding authors and with permission from ICES.
