# Correction: Transformer-based deep learning ensemble framework predicts autism spectrum disorder using health administrative and birth registry data

**DOI:** 10.1038/s41598-025-19777-y

**Published:** 2025-09-29

**Authors:** Kevin Dick, Emily Kaczmarek, Robin Ducharme, Alexa C. Bowie, Alysha L. J. Dingwall-Harvey, Heather Howley, Steven Hawken, Mark C. Walker, Christine M. Armour

**Affiliations:** 1 Better Outcomes Registry & Network (BORN) Ontario, Ottawa, Canada; 2 Prenatal Screening Ontario, Better Outcomes Registry & Network, Ottawa, Canada; 3 Children’s Hospital of Eastern Ontario Research Institute (CHEO-RI), Ottawa, Canada; 4 Clinical Epidemiology Program, Ottawa Hospital Research Institute, Ottawa, Canada; 5 School of Epidemiology and Public Health, University of Ottawa, Ottawa, Canada; 6 Department of Obstetrics and Gynecology, University of Ottawa, Ottawa, Canada; 7 ICES, Toronto, Canada; 8 International and Global Health Office, University of Ottawa, Ottawa, Canada; 9 Department of Obstetrics, Gynecology & Newborn Care, The Ottawa Hospital, Ottawa, Canada; 10 Department of Pediatrics, University of Ottawa, Ottawa, Canada; 11 Department of Genetics, CHEO, Ottawa, Canada

Correction to: *Scientific Reports* 10.1038/s41598-025-90216-8, published online 07 April 2025

The original version of this Article contained an error in Figure 5, where the “Labour Type” variable was attributed to the dataset NSO instead of the BORN BIS dataset. The original Figure 5 and accompanying legend appear below.

**Fig. 5:**
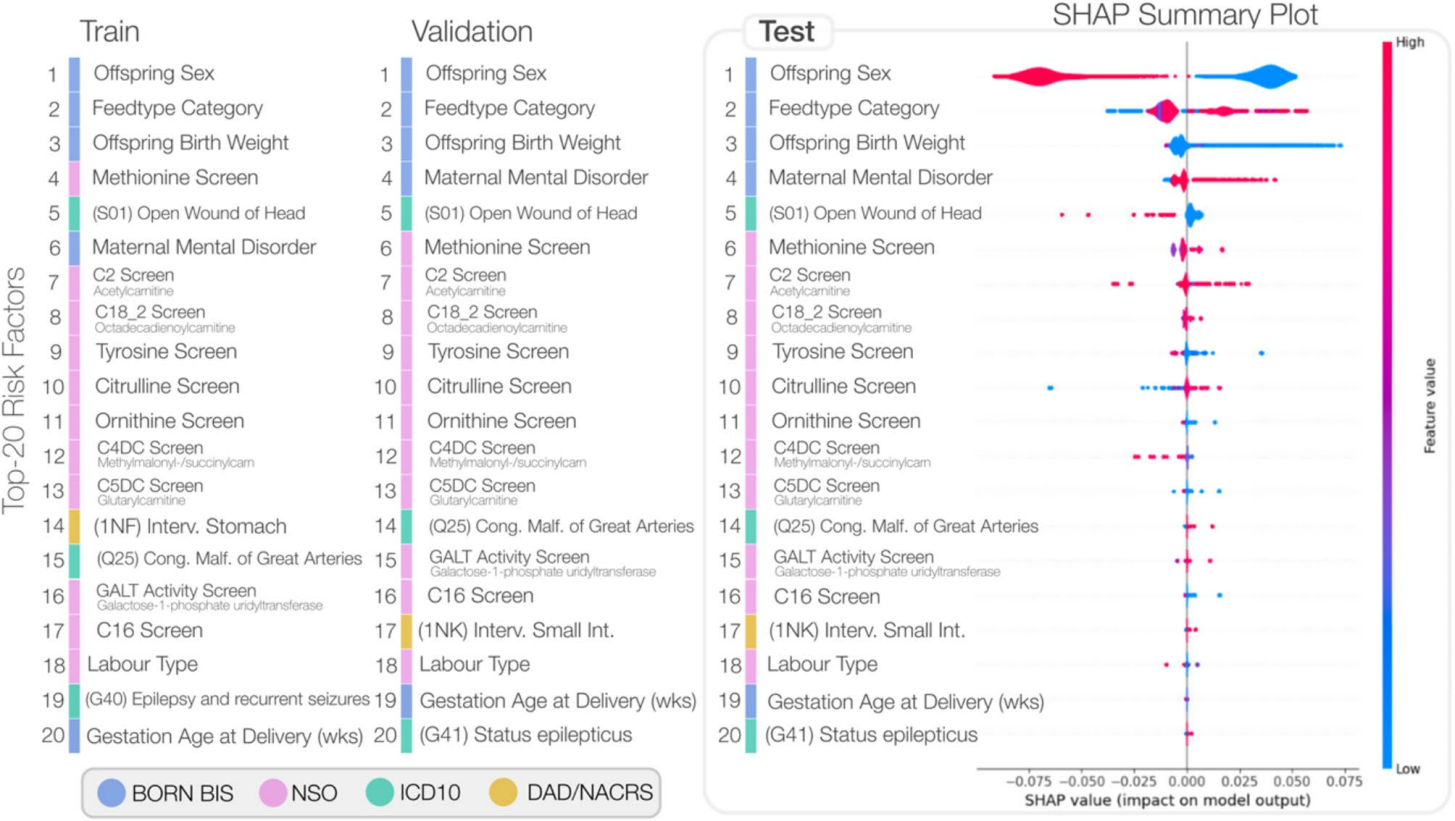
Summary of the top-ranking risk factors determined using SHAP analysis across the three independent datasets. The right-most SHAP summary plot depicts the violin plot distribution for each factor. The possible feature values for each variable are tabulated in Supplementary Table S3; individual KDE plots comparing ASD and controls are illustrated in Supplementary Figure S2. Both the C2 and C16 screen for acetylcarnitine analytes.

The original Article has been corrected.

