# Supplementary material for: Transformer-based deep learning ensemble framework predicts autism spectrum disorder using health administrative and birth registry data
Source: Sci Rep. 2025 Apr 7;15:11816. doi: 10.1038/s41598-025-90216-8 (PMC11977201; doi:10.1038/s41598-025-90216-8)
Supplement: Supplementary file 1 — Supplementary Material 1 [file 41598_2025_90216_MOESM1_ESM.pdf]

Supplementary Appendix

Transformer-Based Deep Learning Ensemble Framework Predicts Autism Spectrum Disorder using Health Administrative & Birth Registry Data

Authors: Kevin Dick[1,2,3,*], Emily Kaczmarek[3], Robin Ducharme[4], Alexa C. Bowie[4], Alysha L.J. Dingwall-Harvey[3,4], Heather Howley[1,2,3], Steven Hawken[3,4,5,6,7], Mark C. Walker[1,3,4,5,6,8,9,10], Christine M. Armour[1,2,3,10,11*]

Affiliations:
1. Better Outcomes Registry & Network (BORN) Ontario, Ottawa, Canada
2. Prenatal Screening Ontario, Better Outcomes Registry & Network, Ottawa, Canada
3. Children's Hospital of Eastern Ontario Research Institute (CHEO-RI), Ottawa, Canada
4. Clinical Epidemiology Program, Ottawa Hospital Research Institute, Ottawa, Canada
5. School of Epidemiology and Public Health, University of Ottawa, Ottawa, Canada
6. Department of Obstetrics and Gynecology, University of Ottawa, Ottawa, Canada
7. ICES, Toronto, Canada
8. International and Global Health Office, University of Ottawa, Ottawa, Canada
9. Department of Obstetrics, Gynecology & Newborn Care, The Ottawa Hospital, Ottawa, Canada
10. Department of Pediatrics, University of Ottawa, Ottawa, Canada
11. Department of Genetics, CHEO, Ottawa, Canada

*** kdick@bornontario.ca; carmour@cheo.on.ca**

Supplementary Methods

1. Temporal Data Preprocessing

The diagnostic and intervention code datasets (DAD, NACRS) were acquired from ICES as large data frames, with each row representing a single hospital/emergency visit. Each diagnosis and intervention code assigned to a patient during a specific visit was separated into an individual column. All infant visits (for ASD cases and non-cases) occurring after 5 years of age were censored, and for ASD cases, the visit with the initial ASD diagnosis was censored, in addition to all visits following the diagnosis date. Across all remaining visits, there were a total of 17,142 unique diagnosis and intervention codes. In preparation for input to BEHRT, DAD and NACRS information for a single patient were concatenated to form a sequence of temporal medical visits. Following terminology defined in BEHRT (Figure 1D), each patient's (*i.e.*, mother or offspring) medical history can be defined as a series of visits, $V_p = \{v_p^1, v_p^2, v_p^3, \ldots v_p^{n_p}\}$, where each patient, p, has a total of n_p visits. Each individual visit, v_p^j, is defined by m number of diagnosis and/or intervention codes c assigned to each patient p for visit j, given by $v_p^j = \{c_p^1, c_p^2, c_p^3 \ldots c_p^{m_j}\}$. For each patient, visits were temporally ordered and concatenated together, with '*SEP*' added between visits to distinguish one from another. Further, we then concatenated mother visit V_{mom} sequences with their respective offspring visit sequences V_{off} to represent visits throughout the entire study timeline, beginning two years prior to birth and ending with the last infant hospital visit before 5 years of age. Similar to BEHRT, we add '*CLS*' at the beginning of the entire sequence. This resulted in a final sequence of $V_{preg} = \{CLS, v_{mom}^1, SEP, \ldots, v_{mom}^{n_{mom}}, SEP, v_{off}^1, SEP, \ldots, v_{off}^{n_{off}}\}$.

To consider temporal information and distinguish between mother and offspring codes, we developed two additional sequences which can be simultaneously input to BEHRT with the code sequence previously described. First, the timeframe between diagnosis and/or intervention codes and their proximity to the birthdate of the offspring may impact ASD diagnosis. Thus, the date of each visit defined in V_{preg} relative to the birth of the infant is used to create a sequence of dates, $D_p = \{d_p^1, d_p^2, d_p^3, \ldots d_p^{n_p}\}$, or $D_{preg} = \{d_{mom}^1, d_{mom}^1, d_{mom}^1, \ldots, d_{mom}^{n_{mom}}, d_{mom}^{n_{mom}}, d_{off}^1, d_{off}^1, \ldots, d_{off}^{n_{off}}\}$ to match the V_{preg} sequence defined above. To match the length of the visit sequence, the date is repeated for each code assigned during that visit, and all '*SEP*' and '*CLS*' values. Therefore, for a single visit, all dates will be identical. Next, while the date sequence implicitly defines if a code is assigned to the mother or offspring (all negative dates correspond to the mother, and all positive dates correspond to the child), we also explicitly assign each visit to a patient. The same diagnosis or intervention code may have higher significance for the mother or offspring, and as such, the patient should be explicitly defined within the input to BEHRT. This sequence consists of single integers to separate one patient from another. In our case, we assign 0 to all visits corresponding to the mother (up to the index date) and switches to 1 for infant visits. As with the date sequence, the values are repeated for all diagnosis and intervention codes, as well as '*SEP*' and '*CLS*' values. Therefore, $P_{preg} = \{0, 0, 0, \ldots, 0, 0, 1, 1, \ldots, 1\}$.

To compare our modified BEHRT time series-based model to the state-of-the-art XGBoost ML architecture, we transformed DAD and NACRS data into static data variables. Rather than one-hot encoding 17,142 individual diagnosis and intervention codes, only a subset of codes were used to define specific health conditions. Specifically, we created variables to define the presence of: maternal diabetes, hypertension, heart disease, asthma, ASD, attention-deficit/hyperactivity disorder, mental health disorder, mood disorder, anxiety disorder, psychotic disorder, or neurodevelopmental disorder diagnosis; smoking, alcohol, or drug use during pregnancy; conception type, mode of delivery, labour type, intervention type during delivery; and/or NICU admission. These variables were also augmented with data from BORN for full completeness. ICD-10 diagnosis codes, CCI intervention codes, and BORN data used to define each variable can be found in Supplementary Data. In addition to these 19 created variables, we also grouped codes based on their first three characters, for a further 2,669 static DAD and NACRS variables.

2. Static Data Preprocessing

2.1 Definitions of Static Variables

Below we list all static variable data definitions in the preparation of the cohort for our study. Where necessary, the original linking dataset fields are listed along with the field identifiers that contribute to the resultant categorical variable leveraged in this work. Where appropriate, the tabulated criteria are formatted as binary logical expressions leveraging <u>AND</u>, <u>OR</u>, <u>NOT</u>, <u>CONTAINS</u> (equiv. "in", '='), <u>DOES NOT CONTAIN</u> (equiv. "≠"), <u>ANY</u> conditions and ()-bracket notation separating clauses to succinctly describe the combination of data processing operations.

2.1.1 – Maternal Characteristics

Maternal Ancestry: Categorical variable that indicates the mother's ancestry.

- (1) First Nation
- (2) East Asian
- (3) Asian

- (4) Black
- (5) Caucasian
- (0) Other
- (-1) Unknown

Marital status: Categorical variable that indicates the mother's ancestry.
- (1) Married/Common-law
- (2) Separated/Divorced
- (3) Single
- (-1) Unknown

Pre-pregnancy BMI : Variable representing the maternal pre-pregnancy BMI (units: kg/m^2), represented both as a numeric and categorical variable.
Calculated within the BORN - BIS: MAT_BMI_NUM = PRE_PREG_MATERNAL_WT_KG ÷ ((MATERNAL_HEIGHT_CM ÷ 100) ^2)
- Continuous: MAT_BMI_NUM
- Categorical, n (%): MAT_BMI_CAT (using MAT_BMI_NUM values)
    - Underweight (BMI <18.5)
    - Normal (BMI 18.5 - < 25)
    - Overweight (BMI 25.0 - <30)
    - Class I Obesity (BMI 30 - <35)
    - Class II Obesity (BMI 35 - <40)
    - Class III Obesity (BMI ≥ 40)
    - Missing

Maternal health condition (any): Categorical variable that indicates if the mother has any pre-existing health condition(s)**.** Excludes mental health problems (*e.g.*, anxiety) substance use, and problems specific to pregnancy (*e.g.*, preeclampsia).

Table S1. MATERNAL_HEALTH variable as a categorical variable extracted from the BIS dataset.

Value	Criteria
YES (1)	MAT_PRE_EXIST_HEALTH_COND_ID ≠ (1017375 [Unknown], 1016520 [None], missing)
NO (0)	MAT_PRE_EXIST_HEALTH_COND_ID = 1016520 [None]
Missing (-1)	MAT_PRE_EXIST_HEALTH_COND_ID = (1017375 [Unknown] or missing)

Maternal diabetes: Categorical variable that indicates if there is a maternal *hx/dx* code for diabetes.

Table S2. MATERNAL_DIABETES as a categorical variable extracted from the BIS dataset.

Value	Criteria
YES (1)	DIABETES_AND_PREGNANCY_ID in (1013400, 1013410, 1013420, 1013430, 1013440, 1013450, 1013460, 1013465, 1013470, 1013480, 1013490, 1013500, 1013510, 1013520, 1013530, 1013540, 1013545, 1013550, 1013560, 1013570, 3000001, 3000002)<br>OR<br>MAT_PRE_EXIST_HEALTH_COND_ID in (1016730, 1016740, 1016750, 1016760, 1016770, 1016780, 3000004)<br>OR<br>INSULIN_DEPENDENT_DIABETES_MELLI = 'YES'

	Diagnosis code for diabetes in DAD/SDS/OHIP/NACRS/ERCLAIMS<br>• OHIP: dxcode in (250)<br>• ICD9 [dxcode] in (250.x, 648.00 – 648.03, 648.8x)<br>• ICD10 [dx10code] in (E10.x, E11.x, E13.x, E14.x, O24.x)
NO (0)	(DIABETES_AND_PREGNANCY_ID = 1013390 or 1013575,missing)<br><u>AND</u><br>(MAT_PRE_EXIST_HEALTH_COND_ID does not contain (1016730, 1016740, 1016750, 1016760, 1016770, 1016780, 3000004, 1017375) or 1017375, missing))<br><u>AND</u><br>(INSULIN_DEPENDENT_DIABETES_MELLI = 'NO' or U, missing)<br><u>AND</u><br><u>NOT</u> (DIABETES_AND_PREGNANCY_ID in (1013575, missing)<br><u>AND</u><br>MAT_PRE_EXIST_HEALTH_COND_ID in (1017375, missing)<br><u>AND</u><br>INSULIN_DEPENDENT_DIABETES_MELLI in (U, missing)<br><u>AND</u><br>• No diagnosis code for diabetes
Missing (-1)	MAT_PRE_EXIST_HEALTH_COND_ID = (1017375 [Unknown] or missing)<br><u>AND</u><br>DIABETES_AND_PREGNANCY_ID = (1013575 [Unknown] or missing)<br><u>AND</u><br>INSULIN_DEPENDENT_DIABETES_MELLI = U or missing<br><u>AND</u><br>• No diagnosis code for diabetes

Maternal hypertension: Categorical variable that indicates if there is a maternal *hx/dx* for hypertension.

Table S3. MATERNAL_HYPERTEN as a categorical variable extracted from the BIS dataset.

Value	Criteria
YES (1)	PREG_HYPERTENSION_DISORDER_ID in (1020800, 1020810, 1020820, 1020840, 1020850, 1020854) <u>OR</u> MAT_PRE_EXIST_HEALTH_COND_ID in (1016660, 1016620)<br><br>Diagnosis code for hypertension* in DAD/SDS/OHIP/NACRS/ERCLAIMS<br>• OHIP: dxcode in (401, 402, 403, 404, and 405, 642)<br>• ICD-9 (dxcode) in (401.x, 402.x, 403.x, 404.x, and 405.x, 642.x)<br>• ICD-10 (dx10code) in (I10.x, I11.x, I12.x, I13.x, I15.x, O10.x, O11.x, O13.x, O14.x, O15.x, O16.x)
NO (0)	PREG_HYPERTENSION_DISORDER_ID = (1020830)<br><u>AND</u><br>MAT_PRE_EXIST_HEALTH_COND_ID in (1016520)<br><u>AND</u><br>• No diagnosis code for hypertension
Missing (-1)	PREG_HYPERTENSION_DISORDER_ID = (1020855, missing)<br><u>AND</u><br>MAT_PRE_EXIST_HEALTH_COND_ID in (1017375, missing)<br><u>AND</u><br>• No diagnosis code for hypertension

**Note: hypertension diagnosis codes are only validated for non-pregnancy hypertension.*

Maternal Heart Disease: Categorical variable that indicates there is a maternal *hx/dx* of heart disease using diagnosis codes.

Table S4. MATERNAL_HEART as a categorical variable extracted from the BIS dataset.

Value	Criteria
YES (1)	MAT_PRE_EXIST_HEALTH_COND_ID in (1016600, 1016610, 1016620, 1016630, 1016640, 1016650, 1016660, 1016670, 1016680)<br><br>Diagnosis code for heart disease in DAD/SDS/OHIP/NACRS/ERCLAIMS<br>• OHIP: dxcode in (390, 391, 394, 398, 402, 410, 412, 413, 415, 426, 427, 428, 429, 745, 746, 747)<br>• ICD-9 (dxcode) in (391.x – 398.x, 402.x – 429.x, 6738.x, 6740.x, 745.x – 747.40, 747.48, 747.49)<br>• ICD10 (dx10code) = (I01.x, I020, I05.x – I52.x (excluding I10.x, I11.x, I12.x, I13.x, I15.x; hypertension), Q20.x – Q26.x, O903.x)
NO (0)	MAT_PRE_EXIST_HEALTH_COND_ID does not contain (1016600, 1016610, 1016620, 1016630, 1016640, 1016650, 1016660, 1016670, 1016680, 1017375, missing)<br>AND<br>• No diagnosis code for heart disease
Missing (-1)	MAT_PRE_EXIST_HEALTH_COND_ID = (1017375 OR Missing)<br>AND<br>• No diagnosis code for heart disease

Maternal asthma: Categorical variable that indicates if the mother has a pre-existing diagnosis of asthma.

Table S5. MATERNAL_ASTHMA as a categorical variable extracted from the BIS dataset.

Value	Criteria
YES (1)	MAT_PRE_EXIST_HEALTH_COND_ID in (1017192, 1017330)<br><br>Diagnosis code for asthma in DAD/SDS/OHIP/NACRS/ERCLAIMS<br>• OHIP: dxcode = 493<br>• ICD-9 (dxcode) = 493.x<br>• ICD-10 (dx10code) = J45.x
NO (0)	MAT_PRE_EXIST_HEALTH_COND_ID does not contain (1017192, 1017330, 1017375, missing)<br>AND<br>• No diagnosis code for asthma
Missing (-1)	MAT_PRE_EXIST_HEALTH_COND_ID = (1017375 or missing)<br>AND<br>• No diagnosis code for asthma

Maternal ASD: Categorical variable to indicate if the mother has a dx of ASD. This variable leverages the same algorithm to identify ASD cases among infants and additionally includes the BORN-BIS indicator for pre-existing ASD condition.

Table S6. MATERNAL_ASD as a categorical variable extracted from the BIS dataset.

Value	Criteria
YES (1)	MAT_PRE_EXIST_HEALTH_COND_ID = 1017250<br><br>Diagnosis code for ASD in DAD/SDS/OHIP/NACRS/ERCLAIMS<br>• OHIP: dxcode = 299<br>• ICD-9 (dxcode) = 299.x<br>• ICD-10 (dx10code) = F84.x
NO (0)	MAT_PRE_EXIST_HEALTH_COND_ID DOES NOT CONTAIN (1017250, 1017375, missing)<br>AND<br>• No diagnosis code for ASD

Missing (-1)	MAT_PRE_EXIST_HEALTH_COND_ID = (1017375 or missing)<br>AND<br>• No diagnosis code for ASD

Maternal Family History of ASD: Categorical variable to indicate if the mother has a family history of ASD.

Table S7. MATERNAL_FAM_ASD as a categorical variable extracted from the BIS dataset.

Value	Criteria
YES (1)	MAT_FAM_HEALTH_HIST_ID = 1022600 [Autism]
NO (0)	MAT_FAM_HEALTH_HIST_ID does not contain (1022600, 1022820, missing)
Missing (-1)	MAT_FAM_HEALTH_HIST_ID = (1022820 [Unknown] or missing)

Maternal Mental Health (any): Categorical variable to indicate ANY maternal hx/*dx* of mental health disorders.

Table S8. MATERNAL_MENTAL as a categorical variable extracted from the BIS dataset.

Value	Criteria
YES (1)	MENTAL_HEALTH_CONCERN_ID in (1017630, 1017640, 1017650, 1017660, 1017670, 1017680, 1017690)<br><br>Diagnosis code for any mental health disorder in DAD/SDS/OHIP/NACRS/ERCLAIMS<br>• OHIP: dxcode in (295-298, 300, 301, 302, 306, 309, 311, 303, 304, 291, 292, 299, 307, 313, 314, 315)<br>• ICD-9 (dxcode) in (291.x, 292.x, 293.x, 295.x-299.x, 300.x – 319.x)<br>• ICD-10 (dx10code) in (F06.x – F99.x) or secondary diagnosis field = X60-X84, Y10-Y19, Y28 when primary dx is not F06-F99
NO (0)	MENTAL_HEALTH_CONCERN_ID == 1017625<br>AND<br>• No diagnosis code for a mental health disorder
Missing (-1)	MENTAL_HEALTH_CONCERN_ID = (1017695, missing)<br>AND<br>• No diagnosis code for a mental health disorder

Maternal Mood Disorder: Categorical variable to indicate maternal *hx/dx* of a mood disorder (includes depression and bipolar disorder) using previously used categories of mental health disorders.

Table S9. MATERNAL_MOOD as a categorical variable extracted from the BIS dataset.

Value	Criteria
YES (1)	MENTAL_HEALTH_CONCERN_ID in (1017660 [Depression], 1017650 [Bipolar])<br><br>Diagnosis code for mood disorder in DAD/SDS/OHIP/NACRS/ERCLAIMS<br>• OHIP: dxcode in (296, 311)<br>• ICD-9 (dxcode) in (293.83, 296.x (all 296 codes), 300.4x, 301.13, 311.x, 625.4)<br>• ICD-10 (dx10code) in (F06.3, F30.x-F34.x, F38.x, F39x, F33.0)
NO (0)	MENTAL_HEALTH_CONCERN_ID does not contain (1017650, 1017660, 1017695, missing)<br>AND<br>• No diagnosis code for a mood disorder
Missing (-1)	MENTAL_HEALTH_CONCERN_ID = (1017695, missing)<br>AND<br>• No diagnosis code for a mood disorder

Maternal Anxiety Disorder: Categorical variable to indicate maternal *hx/dx* of an anxiety disorder using previously used categories of mental health disorders

Table S10. MATERNAL_ANXIETY as a categorical variable extracted from the BIS dataset.

Value	Criteria
YES (1)	MENTAL_HEALTH_CONCERN_ID = 1017640<br><br>Diagnosis code for anxiety disorder in DAD/SDS/OHIP/NACRS/ERCLAIMS<br>• OHIP: dxcode = 300<br>• ICD-9 (dxcode) in (293.84, 300, 300.0x, 300.2x, 309.21, 313.23)<br>• ICD-10 (dx10code) in (F06.4, F40.x, F41.x, F93.0-2, F94.0)
NO (0)	MENTAL_HEALTH_CONCERN_ID not in (1017640, 1017625, 1017695, missing)<br>AND<br>• No diagnosis code for an anxiety disorder
Missing (-1)	MENTAL_HEALTH_CONCERN_ID = (1017695 [Unknown], missing)<br>AND<br>• No diagnosis code for an anxiety disorder

Maternal Psychotic Disorders: Categorical variable to indicate maternal *hx/dx* of a psychotic disorder (including schizophrenia)

Table S11. MATERNAL_PSYCHOTIC as a categorical variable extracted from the BIS dataset.

Value	Criteria
YES (1)	MENTAL_HEALTH_CONCERN_ID = 1017690<br><br>Diagnosis code for psychotic disorder in DAD/SDS/OHIP/NACRS/ERCLAIMS<br>• OHIP: dxcode in (295, 296, 297, 298)<br>• ICD-9 (dxcode) in (293.81/82, 295.x (all 295 codes), 297.x (all 297 codes), 298.x (all 298 codes)<br>• ICD-10 (dx10code) in (F06.0-2, F20.x, F22.x–F29.x, F53.1)
NO (0)	MENTAL_HEALTH_CONCERN_ID does not contain (1017690, 1017695, missing)<br>AND<br>• No diagnosis code for a psychotic disorder
Missing (-1)	MENTAL_HEALTH_CONCERN_ID = (1017695 [Unknown] or missing)<br>AND<br>• No diagnosis code for a psychotic disorder

Maternal Neurodevelopmental Disorder (any): Categorical variable to indicate ANY maternal *hx/dx* of a neurodevelopmental disorder (including ADHD, ASD, and learning disabilities) based on the DSM-5 classification of Neurodevelopmental Disorders.

Table S12. MATERNAL_NEURO as a categorical variable extracted from the BIS dataset.

Value	Criteria
YES (1)	MAT_PRE_EXIST_COND_ID in (1017240, 1017250, 1017260, 1017270, 1017290, 1017310)<br><br>Diagnosis code for neurodevelopmental disorder in DAD/SDS/OHIP/NACRS/ERCLAIMS<br>• OHIP: dxcode in (299, 314, 315, 319)<br>• ICD-9 (dxcode) in (2990, 29900, 29901, 3072, 30720, 30721, 30722, 30723, 3073, 3140, 31400, 31401, 3150, 3151, 3152, 3153, 3154, 3158, 3159, 31500, 31501, 31502, 31509, 31531, 31539, 317, 3170, 3180, 3181, 3182, 319, 3190)

	• ICD-10 (dx10code) in (F70.x, F71.x, F72.x, F73.x, F78.x, F79.x, F80, F80.0-F80.2, F80.8, F80.9, F81.x, F82, F84.x, F88, F88, F89, F90.x, F95.x)
NO (0)	MAT_PRE_EXIST_HEALTH_COND_ID not in (1017240, 1017250, 1017260, 1017270, 1017290, 1017310, 1017375, missing)<br>AND<br>• No diagnosis code for a neurodevelopmental disorder
Missing (-1)	MAT_PRE_EXIST_HEALTH_COND_ID in (1017375, missing)<br>AND<br>• No diagnosis code for a neurodevelopmental disorder

2.1.2 - Delivery/Pregnancy Characteristics

Parity: Categorical variable of parity, PARITY_CAT with the following values:
- 0
- 1
- 2
- 3 +

Number of Fetuses: Categorical variable NUM_FETUS of the number of fetuses in the current pregnancy (BORN – BIS variable NUMBER_OF_FETUSES) with values:
- 1 = 1 (Singleton)
- 2 = 2 (Twin)
- 3+ = 3 or more (*e.g.*, Triplets, Quadruplets, etc.)

Self-reported smoking in pregnancy: Categorical variable indicating whether the birthing individual self-reported having smoked during the pregnancy.

Table S13. SMOKING_IN_PREGNANCY as a categorical variable extracted from the BIS dataset.

Value	Criteria
YES (1)	MAT_SMOKING_AT_ADM_FOR_BIRTH_ID in (1017390, 1017400, 1017410, 1017420)<br>OR<br>MATSMOKINGATFIRSTPRENVISIT_ID in (1017440, 1017450, 1017460, 1017470)<br><br>Diagnosis code for smoking in DAD/SDS/OHIP/NACRS/ERCLAIMS<br>• ICD-10 (dx10code) in (Z716, Z720, F170-173, F179) if recorded during pregnancy (calculated using gestational age at delivery)
NO (0)	(MAT_SMOKING_AT_ADM_FOR_BIRTH_ID = (1017380)<br>AND<br>MATSMOKINGATFIRSTPRENVISIT_ID not in (1017440, 1017450, 1017460, 1017470))<br>OR<br>(MATSMOKINGATFIRSTPRENVISIT_ID = (1017430)<br>AND<br>MAT_SMOKING_AT_ADM_FOR_BIRTH_ID not in (1017390, 1017400, 1017410, 1017420))
Missing (-1)	MAT_SMOKING_AT_ADM_FOR_BIRTH_ID = (1017425, missing)<br>AND<br>MATSMOKINGATFIRSTPRENVISIT_ID = (1017475, missing)

Self-reported drug use in pregnancy: Categorical variable indicating whether the birthing individual self-reported having used drugs (including cannabis) during the pregnancy.

Table S14. MATERNAL_DRUG as a categorical variable extracted from the BIS dataset.

Value	Criteria
YES (1)	EXPOS_DRUG_AND_SUBST_ID in (1020460, 1020470, 1020480, 1020490, 1020500, 1020510, 1020520, 1020530)<br>OR<br>CANNABIS_EXPOSURE_FLAG = [value equivalent to 'Yes']<br><br>Diagnosis code for smoking in DAD/SDS/OHIP/NACRS/ERCLAIMS<br>• ICD-10 (dx10code) in (O35501, O35503, P042, P044, P961) OR (F11.x-F19.x, R78.1-5, R78.8-9, Z72.2, Z50.3, Z71.5-6 if recorded during pregnancy (calculated using gestational age at delivery))
NO (0)	EXPOS_DRUG_AND_SUBST_ID = 1020457<br>AND<br>CANNABIS_EXPOSURE_FLAG = [value equivalent to 'No']
Missing (-1)	EXPOS_DRUG_AND_SUBST_ID = (1020535 or missing)

Self-reported alcohol use in pregnancy: Categorical variable indicating whether the birthing individual self-reported having consumed alcohol during the pregnancy.

Table S15. MATERNAL_ALCOHOL as a categorical variable extracted from the BIS dataset.

Value	Criteria
YES (1)	PREG_EXPOS_ALCOHOL_ID in (1020400, 1020410, 1020420, 1020430, 1020440, 1020442, 1020444, 1020450)<br><br>Diagnosis code for smoking in DAD/SDS/OHIP/NACRS/ERCLAIMS<br>• ICD-10 (dx10code) in (O35401, O35403, O35409, P043, Q860) OR (F10.x, R78.0, T51, Y90.x, Y91.1, Z72.1, Z50.2, Z71.4, X45, X65, Y15 if recorded during pregnancy (calculated using gestational age at delivery))
NO (0)	PREG_EXPOS_ALCOHOL_ID = 1020390
Missing (-1)	PREG_EXPOS_ALCOHOL_ID in (1020455, missing)

Conception type: Categorical variable of spontaneous vs. assisted conception type.

Table S16. CONCEPTION_TYPE as a categorical variable extracted from the BIS, DAD, and NACRS datasets.

Value	BORN-BIS Criteria	DAD/NACRS ICD10 Codes
Spontaneous (1)	CONCEPTION_TYPE_ID = 1013160	Any of (Z37000, Z37200, Z372300, Z37500, Z37510, Z37520, Z37530, Z37580, Z37590, Z37600, Z37610, Z37620, Z37630, Z37680, Z37690, Z37900, Z37910, Z38000, Z38010, Z38100, Z38200, Z38300, Z38310, Z38400, Z38500, Z38600, Z38610, Z38620, Z38630, Z38640, Z38650, Z38660, Z38670, Z38680, Z38690, Z38700, Z38800
Assisted (0)	CONCEPTION_TYPE_ID in (1013110, 1013120, 1013130, 1013140, 1013150, 3000006)	Any of (Z311, Z312, Z313, Z37001, Z37101, Z37201, Z37301, Z37501, Z37511, Z37521, Z37531, Z37581, Z37591, Z37601, Z37611, Z37621, Z37631, Z37681, Z37691, Z37901, Z37911, Z38001, Z38011, Z38101, Z38201, Z38301, Z38311, Z38401, Z38501, Z38601, Z38611, Z38621, Z38631, Z38641, Z38651, Z38661, Z38671, Z38681, Z38691, Z38701, Z38801
Missing (-1)	CONCEPTION_TYPE_ID = (1013180 or missing)	N/A

Mode of delivery: Categorical variable of mode of delivery.

Table S17. MODE_TYPE as a categorical variable extracted from the BIS, DAD, and NACRS datasets.

Value	BORN: BIS Criteria	DAD/NACRS Intervention Codes
Vaginal (1)	BIRTH_TYPE_ID in (1012880, 1012910, 1012920)	Any of (5MD16.xx, 5MD40.xx, 5MD45.xx, 5MD47, 5MD47GJ, 5MD47GK, 5MD47GL, 5MD47GU, 5MD50.xx, 5MD51.xx, 5MD52.xx, 5MD53.xx, 5MD54.xx, 5MD55.xx, 5MD56.xx
Caesarean Section (0)	BIRTH_TYPE_ID in (1012890, 1012900)	Any of (5MD60.xx)
Missing (-1)	BIRTH_TYPE_ID = (1012925 or missing)	N/A

Type of Labour: Categorical variable of the type of delivery.

Table 18. LAB_TYPE as a categorical variable extracted from the BIS, DAD, and NACRS datasets.

Value	BORN: BIS Criteria	DAD/NACRS Intervention Codes
Induced (1)	LABOUR_TYPE_ID = 1014630	Any of (5AC30.xx, 5AC30.xxxx, 5LD31.xx, 5LD31.xxxx)
Spontaneous (2)	LABOUR_TYPE_ID = 1014640	Any of (5MD51.xx)
No Labour (3)	LABOUR_TYPE_ID = 1014645	N/A
Missing (-1)	LABOUR_TYPE_ID = Missing	N/A

Intervention Type: Categorical variable indicating the type of delivery intervention.

Table S19. INTERVENTION_TYPE as a categorical variable extracted from the BIS, DAD, and NACRS datasets.

Value	BORN: BIS Criteria	DAD/NACRS Intervention Codes
Forceps (1)	FORCEPS_VACUUM_ID = 1013830	Any of (5MD53.xx)
Vacuum (2)	FORCEPS_VACUUM_ID = 1013850	Any of (5MD54.xx)
Both (3)	FORCEPS_VACUUM_ID = 1013860	Any of (5MD55.xx)
None (-1)	FORCEPS_VACUUM_ID is not in (1013830, 1013850, 1013860)	No evidence of any intervention used

Pain Management: Categorical variable indicating the type of pain management.

Table S20. PAIN_TYPE as a categorical variable extracted from the BIS dataset.

Value	Criteria
Neuraxial anesthesia (1)	PAIN_MANAGEMENT_LABOUR_BIRTH_ID in (1019820, 1019910, 1019920) OR CS_ANESTHESIA_METHOD_ID in (1013190, 1013200, 1013210)
Other (2)	PAIN_MANAGEMENT_LABOUR_BIRTH_ID in (1019830, 1019840, 1019850, 1019860, 1019870, 1019880, 1019885, 1019890, 1019900) and not in (1019820, 1019910, 1019920)
None (0)	PAIN_MANAGEMENT_LABOUR_BIRTH_ID = 1019810
Missing (-1)	PAIN_MANAGEMENT_LABOUR_BIRTH_ID = (1019930 or missing) AND CS_ANESTHESIA_METHOD_ID = (1013220 or missing)

Maternal intention to breastfeed: Categorical variable representing maternal intention to breastfeed.

Table S21. INTENT_BREASTFEED as a categorical variable extracted from the BIS dataset.

Value	Criteria
YES (1)	INTENTION_TO_BREASTFEED_ID in (1027090, 1027091, 1027092)
NO (0)	INTENTION_TO_BREASTFEED_ID in (1027094)
Missing (-1)	INTENTION_TO_BREASTFEED_ID in (1027096, 3100001) OR Missing

2.1.3 - Offspring Characteristics

Birth season: Categorical variable determined using solstices and equinoxes as the bounds of the season categories (based on delivery date from the MOMBABY dataset, field B_BDATE).

Table S22. BIRTH_SEASON as a categorical variable extracted from the MOMBABY dataset.

Value	Criteria
Spring (1)	B_BDATE in MAR 20 & ≤ JUN 19
Summer (2)	B_BDATE in JUN 20 & ≤ SEPT 21
Fall (3)	B_BDATE in SEPT 22 & ≤ DEC 20
Winter (4)	B_BDATE in DEC 21 & ≤ MAR 19

Birthweight (grams, g): Numerical variable extracted from the MOMBABY dataset from field B_WEIGHT, measured in grams.

Gestational age at delivery (weeks): Numerical variable extracted from the MOMBABY dataset from field B_GESTWKS_DEL, measured in weeks.

Infant sex: Categorical variable representing sex assignment at birth (either Male or Female) from the MOMBABY dataset, field B_SEX.

Apgar score at 5 min: Categorical variable to identify the proportion of infants with a low (≤ 4 @ 5 minutes) and normal (>4 @ 5 minutes) Apgar score.

Table S23. APGAR_SCORE as a categorical variable extracted from the BIS dataset.

Value	Criteria
Low (1)	APGAR05_SCORE ≤ 4
Normal (0)	APGAR05_SCORE > 4
Missing (-1)	APGAR05_SCORE = Missing

NICU admission: Categorical variable of infants who were admitted to the NICU at any point after delivery.

Table S24. NICU_ADMIT as a categorical variable extracted from the BIS dataset.

Value	Criteria
YES (1)	NICU_ADMISSION_FLAG = YES<br>OR<br>BC_NEONATALTRANSFER_ID in (1018320, 1018330)
NO (0)	NICU_ADMISSION_FLAG = NO<br>OR<br>BC_NEONATALTRANSFER_ID = 1018310
Missing (-1)	NICU_ADMISSION_FLAG = Missing<br>AND<br>BC_NEONATALTRANSFER_ID = Missing

NICU length of stay (hours): Categorical variable indicating the length of stay within the NICU, measured in hours, with the following values:

- 1 = ≥ 24 hours
- 0 = < 24 hours
- -1 = Missing

2.2 Transformations Applied to NSO & PSO

Following the preprocessing of temporal data, we next transformed all static data variables from NSO, PSO, and overall cohort information included in this study. The following transformations were applied to the numeric variables. First, all values were log-transformed; values of zero and under were imputed with half of the lowest non-negative value in the dataset prior to transformation. Next, values were pareto standardized, using the square root of the standard deviation in the denominator. Winsorization was then applied to remove all outliers. Lastly, all missing values were imputed using predictive mean matching using the miceforest package in Python.

Of the 142 available NSO variables, 49 were selected based on low missingness and possible relation to ASD based on clinical judgement, and numeric variables were transformed. We also selected five overall cohort variables: offspring birth season, sex, gestational age, birth weight, and maternal age.

3. Sub-analyses

3.1 Prenatal Screening Ontario Subanalysis

For PSO, there are numerous different prenatal screening tests that can be administered, depending on multiple factors. For this reason, there is a large variation in the completeness of PSO variables limiting the number of samples available to this analysis. To mitigate this, we originally omitted all PSO data from our experiments and instead performed a PSO sub-analysis once the final model was determined. The sub-analysis cohort was limited to the 301,829 pregnancies that received IPS PSO testing. Five numeric PSO variables were chosen and transformed as described above. In addition, four variables were one-hot encoded to define whether an offspring tested positive for trisomy-21, trisomy-18, open neural tube defect, or any rare disorder. The full list of static data variables included can be found in the accompanying DCP. The results of this PSO sub-analysis are available in Table S2where we note that the inclusion/exclusion of PSO variables does not greatly impact the model performance.

3.2 Assessment of Model Performance across Birth Year Strata

To assess how model performance varies across birth year strata, we computed the model performance of the test dataset for each year and report the standard deviation in the performance metrics. Given that the evaluation of the model on a year-specific basis results in a significantly smaller dataset and the model was not optimized specifically for training on specific yearly data to then test on a held-out year (i.e., leave-one-year-out cross-validation), we expect variation in the model performance.

4. Machine Learning Methods

We examined two separate machine learning algorithms for the prediction of ASD: BEHRT, a transformer-based deep learning model [31] developed for analysis of Electronic Health Records (EHR), and Extreme Gradient Boosting (XGBoost) (27).

4.1 - BEHRT

To consider temporal information in the prediction of ASD, we adapted BEHRT [31]. BEHRT is a modification of BERT, a state-of-the-art transformer model designed for natural language processing tasks. Specifically, BERT analyzes sentences (part of larger documents) and learns relationships between words (and their positions in sentences) to understand the context of the sentences. BEHRT uses this same idea to understand relationships in EHR. Modelling codes as words, individual physician/hospital visits as sentences, and entire EHR histories as documents, BEHRT learns relationships between codes to identify how codes are related, understand the context of hospital visits, and predict future codes assigned to a patient. In addition, BEHRT also encodes the age of a patient at the time of an EHR visit to determine the effect of age on the relationships between codes and the prediction of future EHR visits. We modified BEHRT to encode time relative to offspring birth as opposed to age, and to analyze both mother and offspring diagnosis and intervention codes through our patient sequences P_{preg}.

Our modified BEHRT takes in five individual sequences to understand mom-offspring information: code, date, patient, positional, and segment sequences. As described previously, code sequences represent all diagnosis and intervention codes assigned to a patient during a specific medical visit. Both mother and infant codes were concatenated together. The date and patient sequences represent the date the codes were assigned, and the patient (i.e., mom vs offspring) they were assigned to. As described in BEHRT, positional sequences are used to identify the position of a visit in a patient's entire medical history (*e.g.*, first medical visit in study timeframe vs fifth medical visit). Positional sequences were encoded through a popular representation created by Vaswani *et al.* [38]. Segment sequences explicitly separate one visit from another and alternate between 0 and 1. All date, patient, positional, and segment values are repeated for all codes assigned during a given visit, resulting in five separate, same-length sequences.

The BEHRT architecture is designed as follows. Each individual sequence (*e.g.,* code, date, etc.) is input to an embedding layer, which generates a unique latent representation of the information in the sequence. The embeddings are then added together to create a combined representation of the diagnosis/intervention code, the date it was assigned, who it was assigned to, its position within all visits throughout the study timeline, and which segment it belongs to. This combined representation will be termed a 'code representation' for the remainder of this manuscript. The code representation is then used as input to the transformer attention layers. BEHRT uses self-attention mechanisms to simultaneously update latent representations while determining importance of each code representation for the given task. Detailed information regarding attention mechanisms can be found in the original articles.

4.2 - XGBoost

The concept of gradient boosting machines, initially proposed by Friedman [39,40] serves as the foundation for Extreme Gradient Boosting (XGBoost) models [32]. Similar to Random Forest models, XGBoost models consist of an ensemble of classification and regression trees (CART). These models leverage systems optimization and fundamental machine learning principles; in essence, they maximize computational capabilities, allowing for scalability, portability, and notable accuracy. The fundamental concept behind boosting involves assigning equal initial weights to each sample and iteratively adjusting these weights [39]. In each iteration, a training set is constructed based on the sample weights, where samples with higher weights have a greater likelihood of being included. Subsequently, a decision tree is constructed using this training set. Following each training iteration, samples that were misclassified during training receive increased weights. The models are then weighted based on the influence of the current model on decision-making, as each model can only accurately learn a portion of the samples, rendering them "weak" models [39]. Ultimately, the weighted combination of these weak models forms a robust model with enhanced predictive power.

4.3 - Additional Evaluation Metrics

We report the Pr@50Re (Precision at 50% Recall) and Sp@50Se (Specificity at 50% Sensitivity) for the final model presented within this work. The Pr@50Re metric reports the precision of a model when it achieves 50% recall while the Sp@50Se metric reports the specificity of a model when it reaches 50% sensitivity.

5. Architecture Design and Hyperparameter Tuning

We pre-trained BEHRT with masked-language-modelling (MLM). MLM takes input sequences and masks a certain number of code representations within an entire patient's medical history. The model is then trained to predict which codes are masked. By training the model to identify the missing codes based on other codes within a patient's history, relationships between diagnosis/intervention codes are learned by the model. This provides the model with context for all possible codes across all patients, as opposed to inputting codes with no pre-training (which would effectively be meaningless to the model at the start of training). The learned context may improve performance of predicting ASD by allowing the model to better recognize related codes and how they may be associated with an ASD diagnosis. BEHRT is pre-trained with MLM for 30 epochs with randomly initialized code, date, patient, positional, and sequence embedding weights. We applied the methods outlined in BEHRT [31], selecting 12% of codes to be masked, and 1.5% of codes to be randomly replaced with other codes.

In this study, we used the same architecture determined to have best performance in the original BEHRT article [31]. Specifically, we chose 6 hidden layers with a size of 288, 12 attention heads, an intermediate layer size of 512, a learning rate of 3e-5, and a dropout rate of 0.01. All sequences were truncated to a maximum of 200 codes per pregnancy. After pre-training with MLM, the final layer of the network used for MLM predictions was removed and replaced with a linear layer consisting of a single output to predict ASD vs. non-ASD. Prior to the final prediction layer, all static data variables (*i.e.*, the 19 static DAD/NACRS variables, 49 NSO variables, and offspring birth weight, birth season, gestational age, sex, and maternal age) were concatenated with the output from the BEHRT attention layers. The network was then trained for an additional 10-25

epochs. For ASD prediction, a loss function of binary cross-entropy with logits loss function was used with a learning rate of 3e-5.

We focused on hyperparameter tuning related to reducing class imbalance due to the low prevalence of ASD. Specifically, we varied the amount of upsampling of the minority class using PyTorch's Weighted Random Sampler function. This function randomly samples the training data points with replacement; we set the number of overall samples to the original number of training samples, and used various weighting schemes with higher sampling of the minority class. The random sampling therefore simultaneously upsamples the minority class and downsamples the majority class. We also experimented with equal representation of ASD and non-ASD cases by downsampling non-ASD controls to a total of 7,624.

Finally, for the comparative XGBoost model, following the work of Dick *et al.*, we ran large-scale hyperparameter tuning experiments leveraging high-performance computing infrastructure [33]. Given the large-scale dataset of complex heterogenous data available within this work, we considered a very large hyperparameter space varying the learning rate, maximum tree depth, and the number of estimators for a total of n=6,417 independently trained and evaluated models. To visualize and rank top-performing models, we represent model validation performance metrics as a comprehensive heatmap based on a specific metric of interest. Each of the nine subplots depicts the results keeping the learning rate fixed as we vary the maximum tree depth (x-axis) between [3,18] by increments of 1, the number of estimators (y-axis) between [50,600] by increments of 25. Within each subplot, we highlight the maximum value with a black bounding box and the median value with a white bounding box. All results are normalized to the same colour range where lighter values represent better performing models.

Supplementary Results

Within this section, we present additional experimental results to complement the findings presented in the main text. Notably, the up-/downsampling experiments for individual Transformer models (non-ensemble) are summarized in Table S25. The high sensitivity value achieved from 1:1 downsampling inspired the n=62 component model ensemble transformer model presented in the main text.

Table S25. Hyperparameter tuning Transformer models using up-/downsampling of classes on the validation dataset. Experiments indicate that training upon a balanced dataset through downsampling the majority class leads to the highest model sensitivity.

Experiment	Exp. Param.	Sensitivity	Specificity	Accuracy	AUROC	PPV	F1 Score
Upsampling Minority Class	None	0.00%	100.00%	98.40%	66.90%	0.00%	0.00%
	1:2	0.20%	100.00%	98.40%	65.40%	10.00%	0.40%
	1:5	4.90%	98.60%	97.10%	65.60%	5.20%	5.00%
	1:50	9.80%	94.40%	93.00%	62.80%	2.70%	4.20%
	1:63 (balanced)	8.00%	95.90%	94.50%	63.30%	3.00%	4.30%
	1:120	13.40%	88.70%	87.50%	63.10%	1.90%	3.30%
Downsampling Majority Class	1:1	86.90%	30.40%	31.20%	64.90%	2.00%	3.80%

Furthermore, Table S26 summarises the sub-analysis results when including/excluding the PSO variables. Given that this sub-analysis was only applied to a limited dataset (only a small fraction of cases undergo PSO screening), the performance values can only be compared to each other and cannot be compared to other tabulated metrics. We determine that the inclusion of PSO variables does not lead to an increase in a meaningful increase in performance metrics.

Table S26. Limited dataset sub-analysis of component performance with and without PSO data.

Experiment	Exp. Param.	Sensitivity	Specificity	Accuracy	AUROC	PPV	F1 Score
PSO Variable Subanalysis	With PSO	74.40%	52.70%	53.00%	69.80%	2.70%	5.10%
	Without PSO	73.20%	54.70%	55.00%	70.50%	2.70%	5.30%

Following from the work of Dick *et al* [33], we performed large-scale hyperparameter tuning experiments to train thousands of XGBoost models. Three hyperparameters defining the size and complexity of the XGBoost model were selected and visually summarises by validation dataset recall in Figure S1: the learning rate (LR; individual panels), the number of estimators (y-axis) and the maximum tree depth (x-axis). We note that the highest achievable recall 75.5% is achieved with the smallest value LR=0.001, a maximum tree depth=4 and with n=100 individual estimators. Conceptually, this represents a comparatively small forest of comparatively shallow trees. Conveniently, such a model is also among the fastest for inference time given the relatively low complexity of the overall model.

The temporal analysis of model performance when evaluate across year of birth strata demonstrated variation in performance across years when evaluated on the test dataset. The standard deviation for each metric are reported as 9.84% sensitivity, 13.0% specificity, 12.9% accuracy, 8.01% AUROC, 0.823% PPV, and 1.06% NPV. As described in the discussion of the main text, these findings suggest that future work investigate the impact of temporal bias on the data preprocessing and optimizing the model training to specifically adopt a leave-one-year-out cross-validation approach during training.

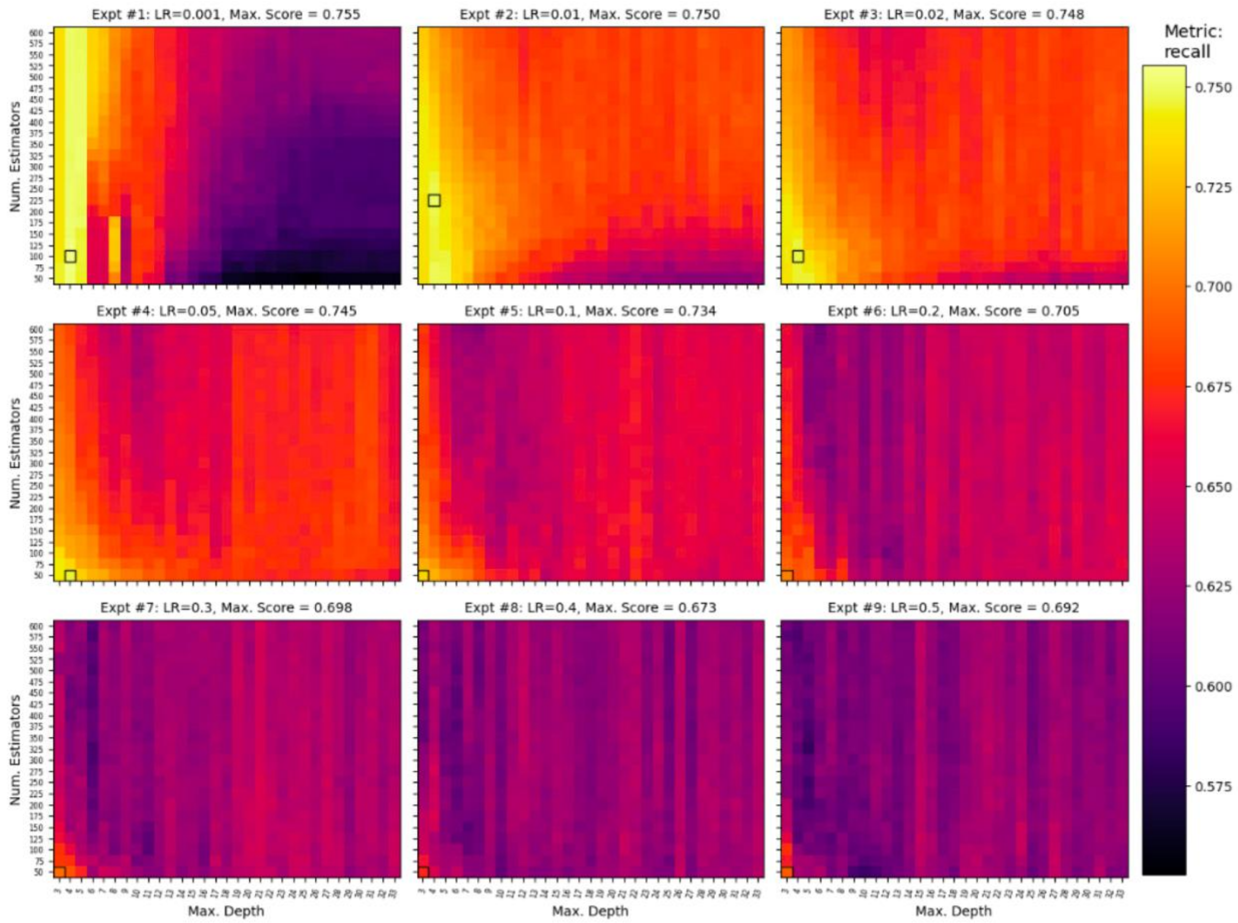

Figure S1. Large-scale hyperparameter tuning experiments of XGBoost models to determine optimal component model configuration. The individual heatmaps each represent one of nine learning rate values, and each varies the maximum tree (x-axis) and the number of component trees (y-axis) of each model. Top-performing models by Recall are identified within each learning rate (LR) panel for inter-panel comparison.

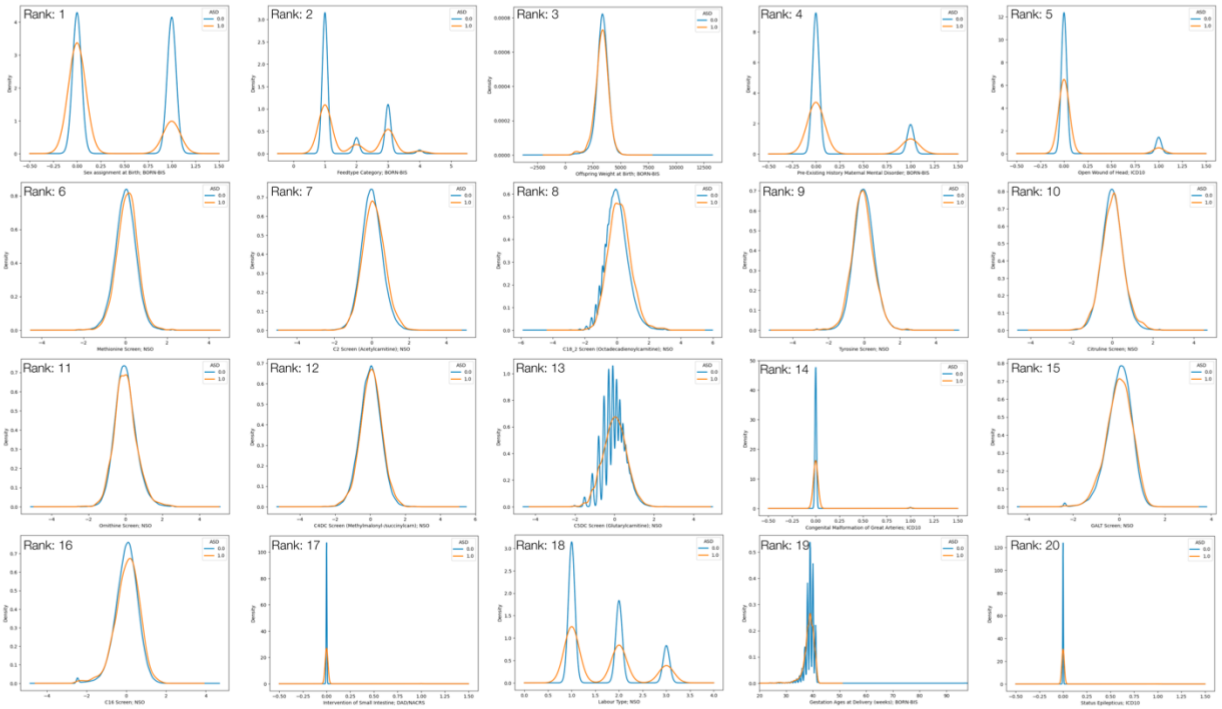

Figure S2. Kernel Density Estimation Plots for the Top-20 Features from top-ranking XGBoost model SHAP Analysis.

Table S27. Tabulation of the top-20 features.

Rank	Variable Id	Variable Name	Dataset	Numeric Values	Category
1	sex	Offspring Sex at Birth	BORN-BIS	0,1	Male, Female
2	feedtype_category	Feedtype Category	BORN-BIS	1,2,3,4	Breast, Formula, Breast and Formula, Other (NPO, TPN)
3	b_weight	Offspring Weight at Birth	BORN-BIS	Continuous	N/A
4	maternal_mental	Maternal Mental Disorder	BORN-BIS	0,1	False, True
5	S01	Open Wound of the Head	ICD10	0,1	False, True
6	met	Methionine Screen	NSO	Continuous	N/A
7	c2	C2 Screen	NSO	Continuous	N/A
8	c18_2	C18_2 Screen	NSO	Continuous	N/A
9	tyr	Tyrosine Screen	NSO	Continuous	N/A
10	cit	Citruline Screen	NSO	Continuous	N/A
11	orn	Ornithine Screen	NSO	Continuous	N/A
12	c4dc	C4DC Screen	NSO	Continuous	N/A
13	c5dc	C5DC Screen	NSO	Continuous	N/A
14	Q25	Congenital Malformation of Great Arteries	ICD10	0,1	False, True
15	galt	GALT Screen	NSO	Continuous	N/A
16	c16	C16 Screen	NSO	Continuous	N/A
17	1NK	Intervention Small Intestines	DAD/NACRS	0,1	False, True
18	lab_type	Labour Type	NSO	1,2,3,-1	Induced, Spontaneous, No Labour, Missing
19	m_gestwks_del	Gestation Age at Delivery	BORN-BIS	Continuous	N/A
20	G41	Status Epilepticus	ICD10	0,1	False, True

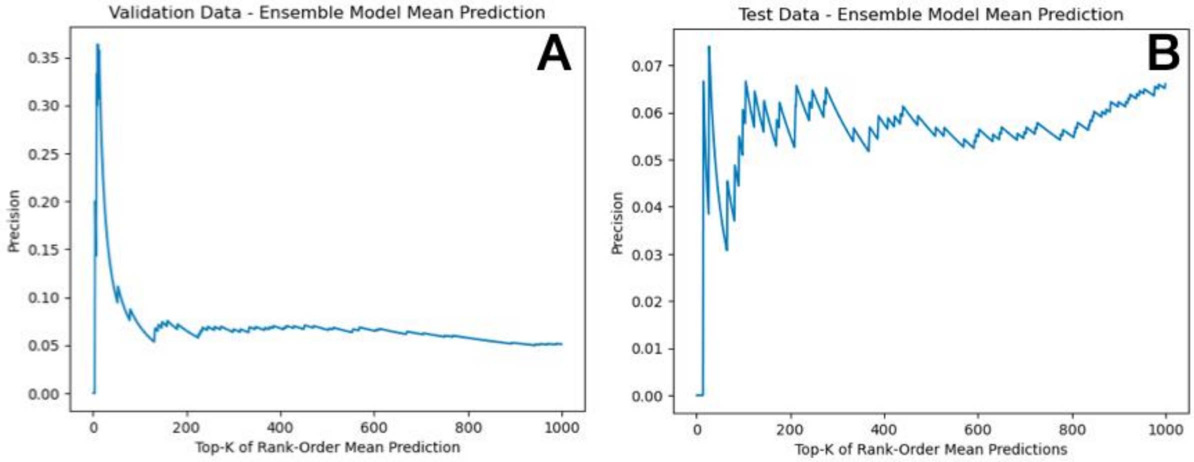

Figure S3. Top-K Precision by Rank Order of Mean Model Prediction score.

References

1. Lord, C., Elsabbagh, M., Baird, G. & Veenstra-Vanderweele, J. Autism spectrum disorder. *The Lancet* **392**, 508–520 (2018).
2. Mehtar, M. & Mukaddes, N. M. Posttraumatic stress disorder in individuals with diagnosis of autistic spectrum disorders. *Res Autism Spectr Disord* **5**, 539–546 (2011).
3. Taylor, J. L. & Gotham, K. O. Cumulative life events, traumatic experiences, and psychiatric symptomatology in transition-aged youth with autism spectrum disorder. *J Neurodev Disord* **8**, 1–11 (2016).
4. Maenner, M. J. *et al.* Prevalence and Characteristics of Autism Spectrum Disorder Among Children Aged 8 Years — Autism and Developmental Disabilities Monitoring Network, 11 Sites, United States, 2018. *MMWR Surveillance Summaries* **70**, 1 (2021).
5. Handleman, J. S. & Harris, S. L. *Preschool Education Programs for Children with Autism*. (Citeseer, 2001).
6. Council, N. R. & others. *Educating Children with Autism*. (National Academies Press, 2001).
7. Dawson, G. *et al.* Randomized, Controlled Trial of an Intervention for Toddlers With Autism: The Early Start Denver Model. *Pediatrics* **125**, e17–e23 (2010).
8. Zwaigenbaum, L. *et al.* Early Screening of Autism Spectrum Disorder: Recommendations for Practice and Research. *Pediatrics* **136**, S41–S59 (2015).
9. Reichow, B. & Wolery, M. Comprehensive synthesis of early intensive behavioral interventions for young children with autism based on the UCLA young autism project model. *J Autism Dev Disord* **39**, 23–41 (2009).
10. Penner, M. *et al.* Community General Pediatricians' Perspectives on Providing Autism Diagnoses in Ontario, Canada: A Qualitative Study. *Journal of Developmental and Behavioral Pediatrics* **38**, 593 (2017).
11. Ip, A. W. S., Zwaigenbaum, L., Nicholas, D. & Sharon, R. Factors influencing autism spectrum disorder screening by community paediatricians. *Paediatr Child Health* **20**, e20–e24 (2015).
12. Rahman, R. *et al.* Identification of newborns at risk for autism using electronic medical records and machine learning. *European Psychiatry* **63**, (2020).
13. Chen, Y.-H., Chen, Q., Kong, L. & Liu, G. Early detection of autism spectrum disorder in young children with machine learning using medical claims data. *BMJ Health Care Inform* **29**, e100544 (2022).
14. Hazlett, H. C. *et al.* Early brain development in infants at high risk for autism spectrum disorder. *Nature* **542**, (2017).
15. Chaitra, N., Vijaya, P. A. & Deshpande, G. Diagnostic prediction of autism spectrum disorder using complex network measures in a machine learning framework. *Biomed Signal Process Control* **62**, (2020).
16. Ahammed, M. S. *et al.* DarkASDNet: Classification of ASD on Functional MRI Using Deep Neural Network. *Front Neuroinform* **15**, (2021).
17. Maenner, M. J., Yeargin-Allsopp, M., Van Braun, K. N., Christensen, D. L. & Schieve, L. A. Development of a machine learning algorithm for the surveillance of autism spectrum disorder. *PLoS One* **11**, (2016).
18. Akter, T. *et al.* Machine Learning-Based Models for Early Stage Detection of Autism Spectrum Disorders. *IEEE Access* **7**, (2019).
19. Omar, K. S., Mondal, P., Khan, N. S., Rizvi, M. R. K. & Islam, M. N. A machine learning approach to predict autism spectrum disorder. in *2019 International conference on electrical, computer and communication engineering (ECCE)* 1–6 (2019).
20. Usta, M. B. *et al.* Use of machine learning methods in prediction of short-term outcome in autism spectrum disorders. *Psychiatry and Clinical Psychopharmacology* **29**, (2019).

21. Vishal, V. *et al.* A Comparative Analysis of Prediction of Autism Spectrum Disorder (ASD) using Machine Learning. in *2022 6th International Conference on Trends in Electronics and Informatics, ICOEI 2022 - Proceedings* (2022). doi:10.1109/ICOEI53556.2022.9777240.
22. Briguglio, M. *et al.* A Machine Learning Approach to the Diagnosis of Autism Spectrum Disorder and Multi-Systemic Developmental Disorder Based on Retrospective Data and ADOS-2 Score. *Brain Sci* **13**, 883 (2023).
23. Oh, D. H., Kim, I. Bin, Kim, S. H. & Ahn, D. H. Predicting autism spectrum disorder using blood-based gene expression signatures and machine learning. *Clinical Psychopharmacology and Neuroscience* **15**, (2017).
24. Voinsky, I., Fridland, O. Y., Aran, A., Frye, R. E. & Gurwitz, D. Machine Learning-Based Blood RNA Signature for Diagnosis of Autism Spectrum Disorder. *Int J Mol Sci* **24**, 2082 (2023).
25. Vaswani, A. *et al.* Attention is all you need. in *Advances in Neural Information Processing Systems* vols 2017-December (2017).
26. Yang, Z., Mitra, A., Liu, W., Berlowitz, D. & Yu, H. TransformEHR: transformer-based encoder-decoder generative model to enhance prediction of disease outcomes using electronic health records. *Nat Commun* **14**, 7857 (2023).
27. Amann, J., Blasimme, A., Vayena, E., Frey, D. & Madai, V. I. Explainability for artificial intelligence in healthcare: a multidisciplinary perspective. *BMC Med Inform Decis Mak* **20**, (2020).
28. Chaddad, A., Peng, J., Xu, J. & Bouridane, A. Survey of Explainable AI Techniques in Healthcare. *Sensors* vol. 23 Preprint at https://doi.org/10.3390/s23020634 (2023).
29. Chen, T. & Guestrin, C. XGBoost: A scalable tree boosting system. in *Proceedings of the ACM SIGKDD International Conference on Knowledge Discovery and Data Mining* vols 13-17-August-2016 (2016).
30. Brooks, J. D. *et al.* Assessing the validity of administrative health data for the identification of children and youth with autism spectrum disorder in Ontario. *Autism Research* **14**, 1037–1045 (2021).
31. Li, Y. *et al.* BEHRT: transformer for electronic health records. *Sci Rep* **10**, 1–12 (2020).
32. Chen, T. *et al.* Xgboost: extreme gradient boosting. *R package version 0.4-2* **1**, 1–4 (2015).
33. Dick, K., Chopra, A., Biggar, K. K. & Green, J. R. Multi-schema computational prediction of the comprehensive SARS-CoV-2 vs. human interactome. *PeerJ* **9**, e11117 (2021).
34. Lundberg, S. M., Allen, P. G. & Lee, S.-I. A Unified Approach to Interpreting Model Predictions. *Adv Neural Inf Process Syst* **30**, (2017).
35. Hemu, A. A. *et al.* Identification of Significant Risk Factors and Impact for ASD Prediction among Children Using Machine Learning Approach. in *2022 2nd International Conference on Advances in Electrical, Computing, Communication and Sustainable Technologies, ICAECT 2022* (2022). doi:10.1109/ICAECT54875.2022.9808043.
36. Bishop-Fitzpatrick, L. *et al.* Using machine learning to identify patterns of lifetime health problems in decedents with autism spectrum disorder. *Autism Research* **11**, 1120–1128 (2018).
37. Betts, K. S., Chai, K., Kisely, S. & Alati, R. Development and validation of a machine learning-based tool to predict autism among children. *Autism Research* **16**, 941–952 (2023).
38. Vaswani, A. *et al.* Attention is All you Need. *Adv Neural Inf Process Syst* **30**, (2017).
39. Friedman, J. H. Greedy function approximation: a gradient boosting machine. *Ann Stat* 1189–1232 (2001).
40. Friedman, J., Hastie, T. & Tibshirani, R. Additive logistic regression: a statistical view of boosting (with discussion and a rejoinder by the authors). *The annals of statistics* **28**, 337–407 (2000).
41. Park, Y. Critical assessment of sequence-based protein-protein interaction prediction methods that do not require homologous protein sequences. *BMC Bioinformatics* **10**, 419 (2009).

42. Dick, K. & Green, J. R. Reciprocal Perspective for Improved Protein-Protein Interaction Prediction. *Sci Rep* (2018).
43. Dick, K. *et al.* Reciprocal perspective as a super learner improves drug-target interaction prediction (MUSDTI). *Scientific Reports 2022 12:1* **12**, 1–19 (2022).
